# A CC‐NB‐ARC‐LRR Gene Regulates Bract Morphology in Cotton

**DOI:** 10.1002/advs.202406111

**Published:** 2024-10-04

**Authors:** Sunyi Yan, Zhanfeng Si, Guoan Qi, Yihao Zang, Lisha Xuan, Lu He, Yiwen Cao, Xiaoran Li, Tianzhen Zhang, Yan Hu

**Affiliations:** ^1^ Zhejiang Provincial Key Laboratory of Crop Genetic Resources Institute of Crop Science Plant Precision Breeding Academy College of Agriculture and Biotechnology Zhejiang University Zhejiang 310058 China; ^2^ Precision Breeding and Germplasm Innovation Team for Cotton and Economic Crops Hainan Institute of Zhejiang University Sanya 572025 China

**Keywords:** auxin, bracts, cottons, nbs‐lrr, ros, single cell transcriptomic landscape

## Abstract

Bracts are leaf‐like structures in flowering plants. They serve multiple functions such as attracting pollinators, aiding tolerance of abiotic stressors, and conducting photosynthesis. While previous studies extensively examine bract function, the molecular mechanisms underlying bract growth remain unknown. Here, the map‐based isolation and characterization of a crucial factor responsible for cotton bract development, identified from a mutant known as frego bract (*fg*), discovered by Frego in 1945 are presented. This gene, named *Ghfg*, encodes a CC‐NB‐ARC‐LRR (CNL) family protein. Through analysis of bract form in plants with virus‐induced gene silencing (VIGS) and transgenic plants, this gene is confirmed to be the causal gene under the *fg* locus. Furthermore, high‐resolution single‐cell transcriptomic landscape of cotton bracts is generated, which reveals differences related to auxin in proliferating cells from TM‐1 and T582; differences in auxin distribution and ROS accumulation are experimentally verified. These findings suggest that GhFG is in a self‐activated state in the *fg* mutant, and its activity leads to ROS accumulation that impacts auxin distribution and transport. Finally, an island cotton variety with the frego bract trait is developed, demonstrating a novel solution for reducing the high impurity rate caused by bract remnants.

## Graphical abstract

This research article focuses on cotton bracts. The authors clone the gene of the frego bract, construct a single‐cell atlas of cotton and excavate genes related to bract type‐related and analyze the mechanism of the different cell morphology of the two bracts. They also elucidate the underlying mechanisms involved in bract development and develop a novel material that is created in island cotton.

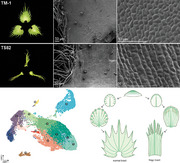

## 1 Introduction

Bracts, also collectively called the calyx, are leaf‐like structures often found beneath a flower or inflorescence. Bracts are common in many plant families and exhibit extensive diversity in color, shape, and other morphological characteristics. In many cases, bracts do not develop synchronously with flowers, which is linked to the role of bracts in relation to flower organs.^[^ ^1^ ^]^ The bracts of some plants are green, like leaves, and perform photosynthesis to provide a source of carbon for seed development. Brightly colored bracts can attract pollinators as well as predators, such as with the creamy yellow bracts of *Rheum nobile* ^[^ ^2^ ^]^ and the pink bracts of *Bougainvillea*.^[^ ^3^ ^]^ Bract shape can also facilitate functions that support the growth of flower organs. In some plants, bracts that enclose flowers help those organs withstand abiotic stress.^[^ ^4^ ^]^ For example, maize bracts enclose the tender seed, protect it from predators, and create a safe and comfortable microenvironment for seed maturation.^[^ ^1^ ^]^ The needle‐like bracts of *Silybum marianum* form a different sort of protective barrier, enabling the fruit to roll on the ground and safeguarding the seeds. Besides providing physical defense, some bracts such as those of *Ficus* emit repellent odors through their stomata, serving as a chemical defense against predators.^[^ ^5^ ^]^

A wild‐type cotton bud is characterized by three large and flat bracts that form a triangle shape to protect the bud against herbivores or adverse abiotic factors. The growth and senescence of these bracts respectively coincide with flower development and boll ripening and opening, with the bract being retained until harvest. As non‐leaf green organs, cotton bracts can also carry out photosynthesis to provide substantial carbon assimilation to the developing bolls.^[^ ^6^ ^]^ When normal leaves are stressed, the photosynthesis contribution rate of bracts increases.^[^ ^7^, ^8^, ^9^ ^]^ Therefore, bracts not only play an important role in boll growth and development but also contribute to stress resistance. However, the microenvironment created by big cotton bracts can also attract boll‐eating pests such as bollworms and boll weevils, who like to hide in the bracts. The boll weevil was once the most destructive agricultural pest in the United States. The weevil invaded from Mexico 100 years ago and wreaked havoc on cotton fields, almost destroying the entire cotton production. In addition to potentially sheltering pests, the bracts also hinder insecticide efficacy. Moreover, withered bracts are a main source of impurities in machine‐picked cotton fiber, especially island cotton, which has larger bracts compared with upland cotton. Given all of these considerations, there is an advantage in cultivating a cotton variety with smaller bracts that do not cover the bolls.

A natural bract mutant called frego bract (*fg*), characterized by slender and twisty bracts, was first discovered in 1945 by a local farmer named Frego in Kansas. This mutant was noticed for its distinct bract morphology that exposes the flower buds, enabling adherence of pesticides to prevent and control pests such as bollworms and boll weevils.^[^ ^10^, ^11^, ^12^ ^]^ Accordingly, *fg* was considered as a source of resistance to boll weevils. Subsequently, Green revealed the genetic characterization of *fg*: in the F_2_ generation of a cross between *fg* and Deltatype webber, normal bracts:frego bracts conformed to a 3:1 segregation ratio, indicating that the inheritance of *fg* is controlled by a single Mendelian recessive gene.^[^ ^13^ ^]^ In 1983, the *fg* locus was mapped to chromosome A03 by Endrizzi.^[^ ^14^ ^]^ Following advancements in upland cotton genome assembly and forward genetic techniques like bulk segregant analysis (BSA‐seq), Zhu of our lab applied BSA‐seq to successfully localize five recessive genes in T582, including *fg*, confirming its mapping to chromosome A03.^[^ ^15^ ^]^ In this study, we further fine‐mapped the *fg* locus and ultimately isolated the candidate gene *Ghfg*, which encodes a CC‐NB‐ARC‐LRR (CNL) family protein with a typical CNL structure.

CNL has predominantly been associated with immune‐related research,^[^ ^16^, ^17^ ^]^ and there remains insufficient understanding regarding the mechanisms by which CNL‐mediated immune responses induce leaf morphological changes. Most known mechanisms involve gain‐of‐function mutants, as CNL is typically self‐inhibited; moreover, some mutations lead to self‐activation.^[^ ^18^ ^]^ For instance, the *Arabidopsis* mutant *snc1* (*suppressor of npr1‐1, constitutive 1*), which displays leaf necrosis and curling, is a gain‐of‐function mutant that results from a point mutation in a non‐domain region of CNL. Upon knockout of the mutant gene, the phenotype of *snc1* reverted to wild‐type.^[^ ^19^ ^]^ Similarly, the rice mutant *nrtp1‐D* has a point mutation in the CNL domain, and its activity leads to a spread of immune reactions such as shortened hypocotyls and roots, curled and narrow leaves, and necrotic spots, and even lethality in homozygous mutants.^[^ ^20^ ^]^ Isolation and characterization of the *Ghfg* gene is not only helpful in advancing our understanding of the morphologic development of cotton bracts but can give insights into the roles of CNL‐mediated immune responses in bracts of other plants.

## 2 Results

### 2.1 Morphological Characteristics of Two Types of Bracts in Cotton

**Figure 1:**
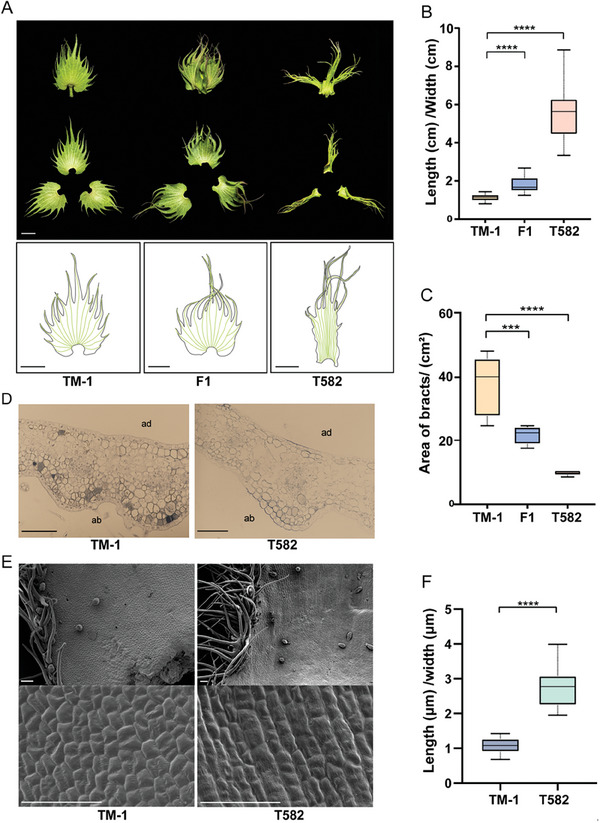
The bract phenotypes of TM‐1 and T582. A) Bract and vein morphology of TM‐1, T582, and F_1_, bar = 10 mm. B,C) Statistics of length/width ratio of bracts and leaf area of three varieties (*n* > 20 each group). D) Paraffin sections of TM‐1 and T582 bract, bar = 100 µm, ad: adaxial, ab: abaxial. E) Morphological difference of epidermal cells in two kinds of bracts, bar = 50 µm. F) Length/width ratio of bract cells (*n* = 40 each group). Statistical analysis was performed using Student's *t*‐test, and significance is denoted as ***p < 0.001, ****p < 0.0001. Data are presented as mean ± s.d.

Texas 582 (T582) is a multiple recessive marker line that carries the *fg* genetic locus from the *fg* mutant (Figure S1A, Supporting Information).^[^ ^21^ ^]^ T582 shares the same genetic background as the genetic standard line of upland cotton, Texas Marker‐1 (TM‐1).^[^ ^22^ ^]^ The bracts of T582 are curled and narrow compared to TM‐1 and do not enclose the flower buds. In F_1_ plants from a cross between T582 and TM‐1, the bract morphology was intermediate with only slight waviness (**Figure** **1**A). Additionally, this unique frego bract reduce the accumulation of residue in fiber during mechanical harvesting (Figure S1B, Supporting Information).

On normal bracts, the serrations are evenly distributed along the margins, and the veins take on a radiating shape that follows their distribution. In contrast, frego bracts have serrations distributed only on the upside, and the veins run in parallel (Figure 1A), resulting in narrower, crumpled bracts (Figure 1B, measurement method in Figure S1C, Supporting Information). Our measurement showed the area of frego bracts to be much smaller, amounting to almost one‐quarter the area of normal bracts (Figure 1C). The two types of bracts also differ in cellular arrangement (Figure 1D): the epidermal cells of normal bracts are neatly arranged, compact, and uniform in size, while those of frego bracts are loosely arranged and have uneven surfaces. Additionally, normal bracts have more distinct mesophyll cell layers than frego bracts. As a general observation, it can also be seen in transverse sections that bract mesophyll cells all have the same morphology, rather than the distinct palisade cells and sponge cells typical of leaves, which means that the mesophyll cells of bracts are not fully differentiated; this reflects the nature of bracts as modified leaves.

We further characterized the epidermal cells of both kinds of bracts by scanning electron microscopy. The frego bracts showed longitudinal cell growth, producing cells with the shape of long strips, and cells at the edge were oriented perpendicular to the bract base. Conversely, the epidermal cells of normal bracts were irregular in shape, cell growth occurred in random directions, and cells at the edge had no obvious pattern (Figure 1E). By calculating the length/width ratio of bract cells, we determined the epidermal cells of frego bracts to have a significantly higher ratio than normal bracts (Figure 1F), consistent with the overall length/width ratios of the two bract types. Based on our morphological observations, we hypothesize that bract longitudinal growth occurs prior to transverse growth, and this is the main factor in producing a long and narrow bract.

### 2.2 Genetic Analysis and Map‐Based Cloning of the *fg* Locus

**Figure 2:**
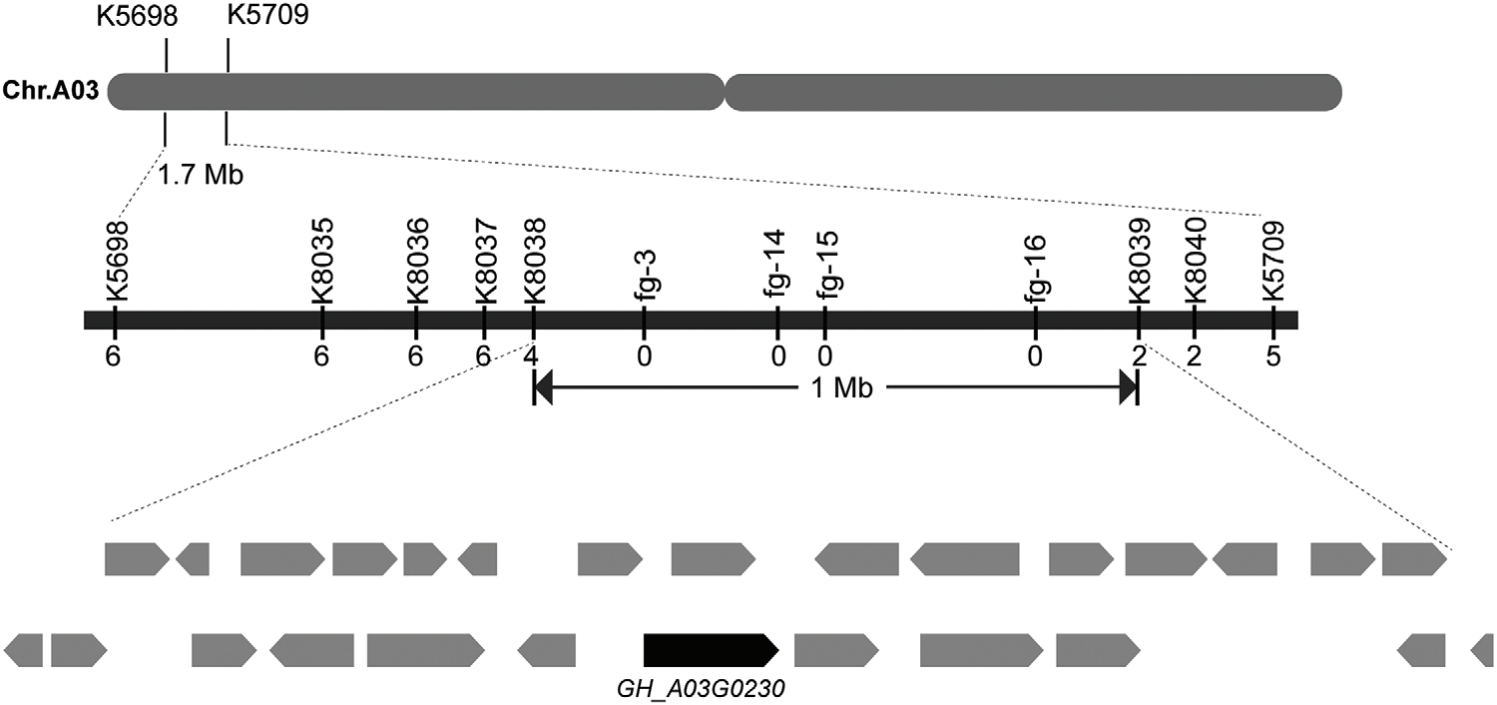
Cloning of the *fg* locus. Using F2 generations of TM‐1 and T582 to locate *fg* in a candidate region of 1‐Mb on chromosome A03 between K8038 and K8039.

Our previous studies^[^ ^15^ ^]^ have shown the trait segregation ratio of normal bracts to frego bracts in F_2_ and BC_1_ mapping populations to be ≈3:1 (AA+Aa:aa) and 1:1 (AA:aa) respectively (F_2_: 1635 normal‐ and 529 frego‐bracted plants, fitting the expected 3:1 ratio: χ2 = 0.3549 < χ2_0.05_ = 3.84, df = 1; BC_1_: 216 normal‐ and 196 frego‐bracted plants, fitting the expected 1:1 ratio: χ2 = 0.97087 < χ2_0.05_ = 3.84, df = 1), indicating that *fg* is controlled by a single recessive gene. Through bulk segregant analysis (BSA‐Seq) of the mutant‐type pool and alignment to the TM‐1 reference genome (TM‐1v1.1)^[^ ^23^ ^]^ the *fg* gene was primarily mapped to a 4.5 Mb interval on chromosome A03. Sequence differences between the two parents were determined within this interval, and InDel primers were designed (Table S1, Supporting Information) using the primer design software PRIMER 3.0. Based on sequencing of those polymorphic markers in 515 F_2_ individuals with the frego bract trait, *fg* was further localized within the candidate interval to between markers K5698 and K5709. Subsequently, single‐nucleotide polymorphism (SNP) primers (Table S1, Supporting Information) were designed; genotyping with these primers further narrowed the candidate interval to between markers K8038 and K8039, which spanned a physical distance of 190.4 kb according to the TM‐1 v1.1 reference genome. Anchoring this interval to the TM‐1 v2.1 reference genome^[^ ^24^ ^]^ expanded its span to 1 Mb (**Figure** **2**), encompassing 878 SNPs and only 1 InDel. Within this expanded interval, there are 76 genes, we used RNA‐seq (Table S2, Supporting Information) data of TM‐1 and T582 bracts to screen out unexpressed genes. There are 27 remaining genes with expression differences, and the locations of these candidate genes are labeled in Figure 2. Based on the resequencing data, we searched for genes with sequence differences, of which only gene *GH_A03G0230* has SNPs and InDel on exons, gene *GH_A03G0209* has SNPs on introns, and genes *GH_A03G0180* and *GH_A03G0232* have SNPs on promoter regions. Moreover, there are nonsynonymous mutations on exons *GH_A03G0230*. We next applied Kompetitive Allele‐Specific PCR (KASP) to the expanded interval to verify the correctness of the map cloning result. Plausibility was confirmed based on the genotypes of 239 frego bract trait strains, and the allelic discrimination plot demonstrated the markers fg‐3, fg‐14, fg‐15, and fg‐16 to co‐segregate with the frego bract trait (Table S3, Supporting Information).

### 2.3 Restoration of Normal Bract Morphology by Silencing of *GH_A03G0230* in fg Mutant Plants

In order to identify the *fg* causative gene from among the candidate genes, we used the RNA‐seq data (Table S2, Supporting Information) to select the plant receptors that should be selected for the Virus‐induced gene sliencing (VIGS) experiment. The RNA‐seq data indicated that the expression level of *GH_A03G0180*, *GH_A03G0209*, *GH_A03G0230*, and *GH_A03G0232* was higher than that of TM‐1 in T582.

**Figure 3:**
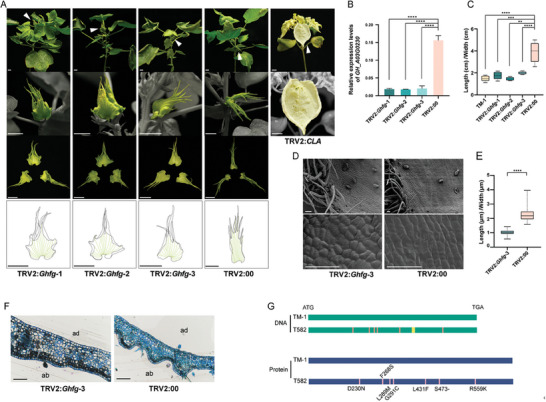
The phenotypes of T582 bracts after VIGS. A) Silencing *Ghfg* led to the frego bracts return to normal, bar = 10 mm. B) qPCR analysis of VIGS silence effect (*n* = 3 biological replicates). C) Length/width ratio of bracts after VIGS. (*n* = 15 each group). D) Morphology of epidermal cells of TRV2:*Ghfg*‐3 and TRV2:00 (*n* = 40 each group), bar = 50 µm. E) Length/width ratio of bracts cells after VIGS (*n* = 15 each group). F) Section of bracts, bar = 100 µm. G) Schematic diagram of DNA sequence and protein sequence of *GH_A03G0230*, orange lines on T582 DNA indicate SNP positions in the *GH_A03G0230*, yellow block indicates InDel position, pink lines indicate the mutant location of amino acids. Statistical analysis was performed using Student's *t*‐test, and significance is denoted as **p < 0.01,***p < 0.001, ****p < 0.0001. Data are presented as mean ± s.d.

VIGS is commonly utilized as a rapid and efficient gene‐silencing strategy^[^ ^25^ ^]^ for functional validation in crops like cotton.^[^ ^26^ ^]^ In our preliminary experiments, except for *GH_A03G0230*, no phenotype appeared after silencing *GH_A03G0180*, *GH_A03G0209*, and *GH_A03G0232*. In view of this, we performed silent verification on *GH_A03G0230* again. As *GH_A03G0230* is a long gene (2526 bp) and includes three domains, we designed three highly specific silencing fragments and used them to construct vectors to knock down the gene in T582 (Table S1, Supporting Information). Observations of the silencing effect were made from ≈50 days after injection when the plants had begun to bud, until the bract was fully expanded. We observed significant changes in the bracts of *GH_A03G0230*‐silenced plants (**Figure** **3**A; Figure S2A, Supporting Information), in that they tended toward more normal morphology, but not in other parts of the plants, such as leaves. qPCR assays confirmed that *GH_A03G0230* was significantly silenced in the bracts of the experimental group (Figure 3B). To quantify changes in the bracts, we measured length and width^[^ ^27^ ^]^ and calculated the ratio for bracts (Figure 3C) and cells (Figure 3D,E). We found the control group (T582) to have a bract length/width ratio of ≈4, while *GH_A03G0230*‐silenced plants had values in between those of TM‐1 and the control group, and gradually approached the trend of TM‐1 bracts, converging to 2. Additionally, there was an observable trend of cells in the bracts of VIGS plants returning to a more organized arrangement (Figure 3F), and the veins to a radial layout. We next sought to further confirm the ability of *GH_A03G0230* silencing to rescue the *fg* phenotype by replicating the silencing in MD51ne plants, which have bracts similar to T582. Following the same VIGS treatment, MD51ne bracts were also restored to normal morphology (Figure S2B,C, Supporting Information). This further supported the silencing of *GH_A03G0230* as sufficient to induce a change in bract morphology, with increased lateral growth reducing the extreme longitudinal growth characteristic of *fg* plants. Hereafter, *GH_A03G0230* is referred to as *Ghfg*.

To determine genomic sequence variation in *Ghfg* among different cotton species, we isolated and compared *Ghfg* sequences from TM‐1 and T582. Comparison of *Ghfg* sequences between the normal bract cottons and the frego bract cotton revealed a total of seven variants (Figure 3G), which may be responsible for the transformation from normal bracts to frego bracts. These variants caused changes in the amino acid sequence: D230N, F268S, L289M, G291C located in the NB‐ARC domain, R559K located in the LRR domain, L431F and InDel S473‐ were between the NB‐ARC and LRR domains.

With the conclusion that frego bract results from point mutations in *Ghfg*, it is necessary to demonstrate that *Ghfg* is self‐activated by such mutations. Accordingly, we constructed overexpression vectors for wild‐type and mutated *Ghfg* driven by the CMV35S promoter, then injected the constructs into tobacco leaves. After 48–72 h, distinct effects were observed: tobacco leaves injected with the mutated constructs exhibited significant necrosis, while those injected with the wild‐type construct showed minimal necrotic symptoms (Figure S3A, Supporting Information). After trypan blue staining, the necrotic cells appeared as blue spots (Figure S3B, Supporting Information). At the same time, confocal microscopy was used to observe the expression of two proteins in tobacco cells (Figure S3C, Supporting Information).

### 2.4 *Ghfg* is Responsible for the *fg* Phenotype in Transgenic Cotton

We further confirmed the causal role of GhFG activation in the *fg* phenotype through transgenic experiments in cotton. To achieve genetically stable transgenic cotton plants, we developed a 35s::*Ghfg*‐GFP construct and used it to transform TM‐1 (normal bract) plants, and further applied CRISPR/Cas9 to generate three *Ghfg* mutants (SE‐5, SE‐9, and SE‐12) on the MD51ne (frego bract) background such that the GhFG protein was terminated early (Figure S4A, Supporting Information). Visually, we observed that bracts of the *Ghfg* mutant lines were restored to normal (Figure S4B,C, Supporting Information). SE‐12 in particular exhibited well‐distributed cells and overall flatness, while the untransformed MD51ne control showed irregular cellular arrangement, uneven cell size, and overall distortion (Figure S4D, Supporting Information). The length/width ratio of bracts decreased from ≈3 in untransformed MD51ne to ≈1.5 in the *Ghfg* mutant lines (Figure S4F, Supporting Information). And the changes of bract cells showed the same trend (Figure S4E–G, Supporting Information). These findings suggest that knocking out of *Ghfg* can return bracts to normal in the MD51ne background, confirming that mutation of *Ghfg* is responsible for the frego bract trait. However, *Ghfg* overexpression in TM‐1 plants did not result in observable changes in bracts morphology. We speculate that the expression level achieved may not have been sufficient to elicit a phenotypic response. Further exploration of this area is needed.

### 2.5 A Single‐Cell Expression Atlas and Transcriptome Analysis Revealed the Possible Mechanism by Which Ghfg Regulates Bract Lateral Expansion

**Figure 4:**
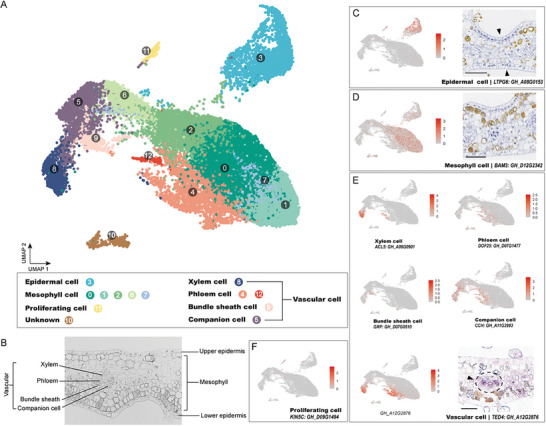
The joint cell clusters of TM‐1 and T582 bracts. A) UMAP visualization of 12 cell clusters. Dots represent cells (*n* = 22985). B) Indication of various tissues on the bract (TM‐1) section. C–F) Position of marker gene on UMAP and RNA in situ hybridization results of some tissues, bar = 50 µm.

To comprehensively characterize the transcriptome of cotton bracts and explore the regulatory network governing bract morphogenesis, we performed single‐cell RNA sequencing (scRNA‐seq) on protoplasts isolated from 15‐day‐old bracts (after squaring). About 700–1000/uL protoplasts were subjected to droplet‐based scRNA‐seq using the 10× Genomics platform (Figure S5, Supporting Information). Cells with low‐count genes, cell‐cycle‐related genes, and doublets were excluded from further analyses. Following quality control (Figures S6–S8, Supporting Information), a total of 10683 cells from TM‐1 and 14649 cells from T582 were analyzed, with the detection of 42089 and 41987 genes respectively (Table S4, Supporting Information). Unsupervised cluster analyses with uniform manifold approximation and projection (UMAP) of the combined data revealed 12 distinct cell clusters (Table S5, Supporting Information). Subsequently, cell identity was assigned to each cluster based on marker genes (Table S6, Supporting Information) previously reported as criteria for clustering (Figures S9,S10, Supporting Information), which grouped the clusters into seven cell populations (**Figure** **4**A, Table S7, Supporting Information): epidermis cells (cluster 3), mesophyll cells (clusters 0, 1, 2, 6, 7), proliferating cells (cluster 11), xylem cells (cluster 8), phloem cells (clusters 4, 12), bundle sheath cells (cluster 9), and companion cells (cluster 5). These groups also correspond to the cell composition of the bracts under a light microscope (Figure 4B).

The epidermal cell cluster is a highly specialized cell group discriminated by specific epidermal cell genes such as *LIPID PROTEIN TRANSFER 6 (LTPG6)* ^[^ ^28^ ^]^ and *DEFECTIVE IN CUTICULAR RIDGES (DCR)*,^[^ ^29^ ^]^ which are closely related to the cuticle on the bract surface (Figure 4C). Mesophyll cells encompass five clusters that are correlated with the photosynthetic pathway, characterized by the expression of genes such as *CARBONIC ANHYDRASE (CAHC1)* ^[^ ^29^ ^]^ and *BETA‐AMYLASE3 (BAM3)* ^[^ ^28^ ^]^ (Figure 4D). Phloem, xylem, and bundle sheath cells are all included in the vascular bundle, which we distinguished based on genes particularly expressed in those three tissues, such as *VASCULAR RELATED NAC‐DOMAIN PROTEIN 1 (VND1)* ^[^ ^29^ ^]^ (Figure 4E). We also employed marker genes specific to the different vascular cells to distinguish between vascular tissues. First, phloem cells were distinguished by expression of the marker gene *DOF AFFECTING GERMINATION 1 (DAG1)*.^[^ ^30^ ^]^ Then, we differentiated the xylem and the bundle sheath, taking the marker genes *ACAULIS 5* and *ABNORMAL SHOOT 5* ^[^ ^31^ ^]^ to determine xylem cells and *GLYCINE‐RICH PROTEIN (GRP)* ^[^ ^28^ ^]^ to determine bundle sheath cells. Finally, proliferating cells were characterized by expression of genes encoding kinesin‐like proteins, such as *KIN5C* ^[^ ^29^ ^]^ (Figure 4F).

We further established a single‐cell atlas of cotton bract and performed Gene Ontology (GO) enrichment analysis for each cell type to confirm their functions (Table S8, Supporting Information). As expected, terms enriched among epidermis cells were mainly related to lipid synthesis and binding (Figure S11A, Supporting Information), while terms in mesophyll cells mostly pertained to photosynthesis (Figure S11B, Supporting Information). Similarly, proliferating cells were enriched in pathways associated with cell proliferation, such as terms relating to the microtubules necessary for cell division (Figure S11C, Supporting Information). Finally, terms enriched in xylem, bundle sheath, and companion cells aligned with the functions of vascular cells, such as processes related to water or metal ion transport (Figure S11D–G, Supporting Information).

**Figure 5:**
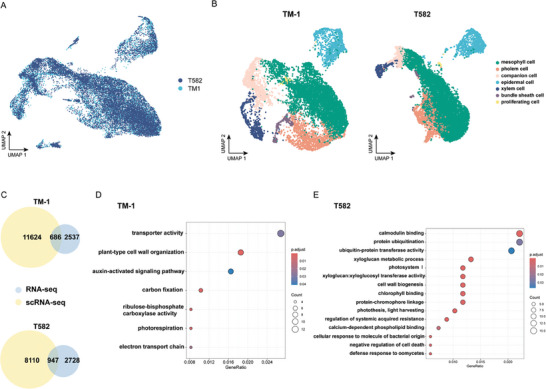
ScRNA‐seq data and RNA‐seq data are combined to analyze DEGs in different cells of TM‐1 and T582 bracts. A) Combined UMAP visualization of TM‐1and T582 scRNA‐seq data after effect removal. B) UMAP visualization shows bract cells were grouped both in TM‐1 and T582. C) Venn diagrams respectively show the up‐regulated expression genes of TM‐1 and T582 in both scRNA‐seq and RNA‐seq data. D,E) Enriched GO terms attributed to genes that were highly expressed in both RNA‐seq and scRNA‐seq of TM‐1 and T582. The color of each dot represents the statistical significance (p. adjust) of the enriched terms, and the diameter of the dot indicates the number of genes associated with each term.

We next used the previously confirmed marker genes to construct single‐cell atlases of TM‐1 and T582 bracts (**Figure** **5**A,B). We also performed RNA‐seq analysis to identify genes that were specifically expressed in bracts of each variety. A gene was considered differentially expressed with the absolute value of *log*_2_FoldChange ≥ 1 and adjusted *P*‐value < 0.05. Venn diagrams were used to identify specific expression of genes in both scRNA‐seq (Table S5, Supporting Information) and RNA‐seq (Table S2, Supporting Information), which yielded 947 T582‐specific genes and 686 TM‐1‐specific genes (Figure 5C). These genes were then subjected to GO enrichment analysis (Figure 5D,E). Genes specific to TM‐1 bracts were enriched for terms such as “auxin‐activated signaling pathway” (GO:0009664) and “plant‐type cell wall organization” (GO:0009734). Genes in the first term included several related to auxin transport and response (*GH_A01G1637, GH_A07G2361, GH_A11G1016, GH_D01G1731, GH_D02G2596, GH_D07G2303, GH_D11G1349*), while the second term included genes annotated as “expansion A4/A15” (*GH_A01G2400, GH_A03G0547, GH_A03G1196, GH_A05G0959, GH_A13G0812, GH_D02G1382, GH_D11G3754, GH_D13G0780*); such proteins often respond to auxin and can cause relaxation of plant cell walls.^[^ ^32^, ^33^ ^]^ These enrichments suggest that TM‐1 and T582 may differ in auxin signal transduction and expansion protein expression, which may influence bract phenotype. In T582 bracts, the analysis highlighted terms like “leaf morphogenesis” (GO:0009965), which includes NAC domain‐containing protein 36 (*GH_A01G0016, GH_A03G2330, GH_D01G0016, GH_D02G2499*), a protein reported to negatively regulate cell size.^[^ ^34^ ^]^ Also enriched was the term “signal transduction calmodulin‐binding” (GO:0005516), which contains genes (*GH_A07G1124, GH_A08G2342, GH_D09G0602*) related to calmodulin receptor regulation or stress response. The results of this enrichment analysis may provide some suggestions for exploring the morphological differences of bracts in further studies.

**Figure 6:**
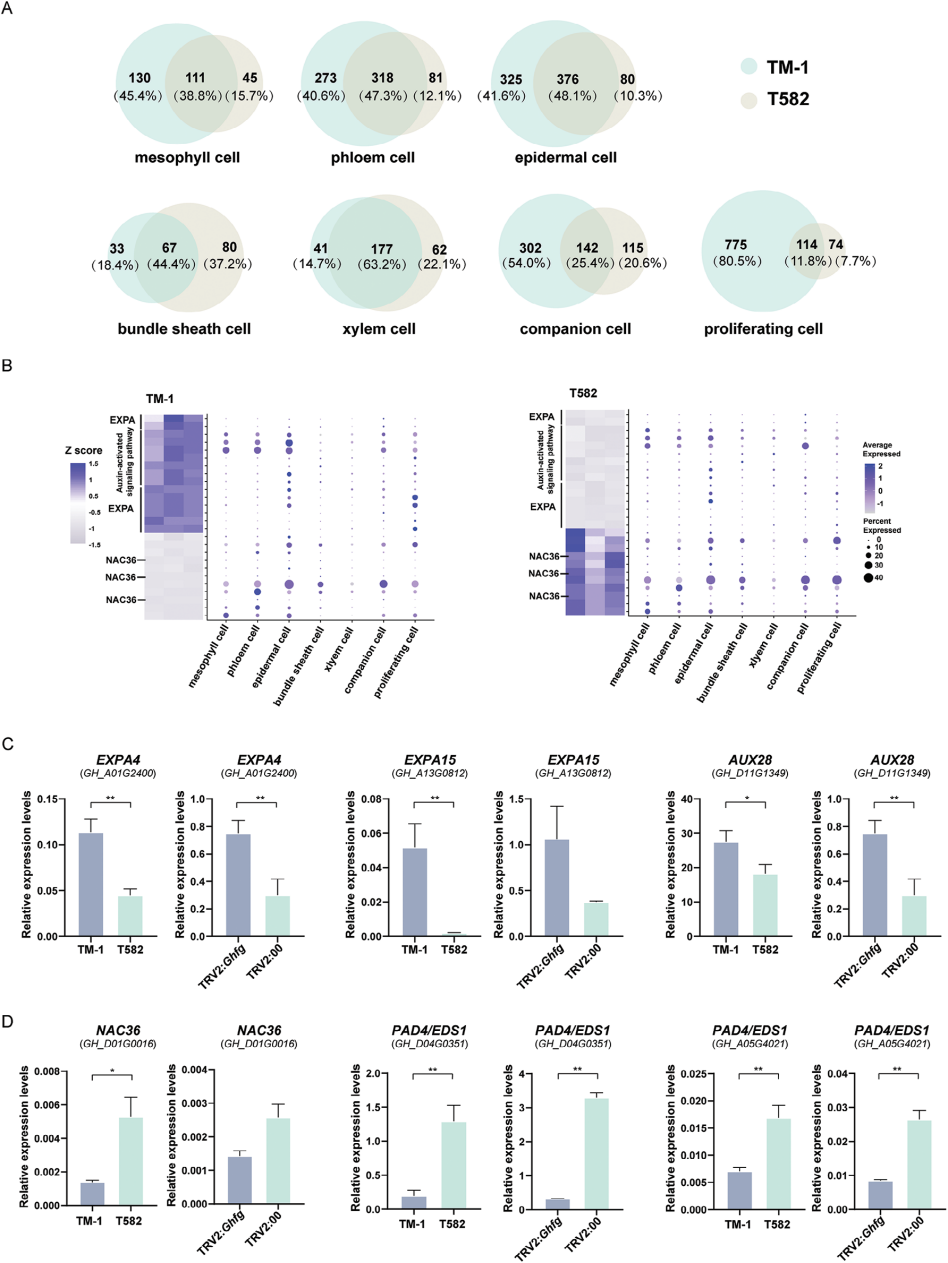
Highly expressed genes in bracts of TM‐1 and T582. A) Venn diagrams show a correspondence between two materials with genes expressed in different cell populations or genes specific to one of the materials. B) Expression patterns of plant‐type cell wall organization, auxin, and leaf morphogenesis pathway genes for scRNA‐seq and RNA‐seq. The dot plots show the expression levels and sits of each gene, the color and size of each dot represents the expression levels, while the *x*‐axis represents the site of gene expression. The heatmaps show the expression of these genes (TPM) in RNA‐seq data. C,D) Quantitative verification of genes with high and low expression in the bracts of TM‐1 and T582 (*n* = 3 biological replicates). *EXPA*, *expansion A4/A15*; *IAA11*, *indole‐3‐acetic acid inducible 11*, *NAC36*, *NAC domain‐containing protein 36*, *PAD4/EDS1*, *alpha/beta‐Hydrolases superfamily protein/enhanced disease susceptibility 1*. Student's *t*‐test and significance is denoted as *p < 0.05,**p < 0.01. Data are presented as mean ± s.d.

Subsequently, we constructed Venn diagrams to examine differentially expressed genes at the cluster level (**Figure** **6**A). This revealed that individual clusters differ significantly in the fraction of co‐expressed genes. Particularly, in the proliferating cells cluster, 114 genes were found to be common in the two varieties and were subsequently removed to enrich for variety‐specific genes (Figure S12A,B, Supporting Information). In TM‐1, genes expressed in the proliferating cells were enriched for terms related to cell division, such as “microtubule binding” (GO:0008017), “microtubule motor activity” (GO:0003777), “cytokinesis” (GO:0000910), and “cell wall organization” (GO:0071555); this is consistent with the characteristics of proliferating cells. Interestingly, that cluster was also enriched for several terms related to auxin, such as “auxin homeostasis” (GO:0010252) (*GH_A05G1432*), “auxin‐activated signaling pathway” (GO:0009734) (*GH_A10G0453, GH_A08G1389, GH_A08G2188, GH_A10G2265*), and “response to auxin” (GO:0009733) (*GH_D02G1906/ GH_D08G2790*); this further highlights auxin as a point of difference between TM‐1 and T582, particularly in proliferating cells. Meanwhile, proliferating cells in T582 exhibited enrichment of terms associated with oxidation and antioxidants, such as “oxidoreductase activity” (GO:0016620) (*GH_A03G1495, GH_D02G1668*), “NADP binding” (GO:0050661) (*GH_A03G1495, GH_D02G1668*), and “NAD binding” (GO:0051287) (*GH_A03G1495,GH_D02G1668*). These findings are consistent with the enrichments observed for genes differentially expressed between *Ghfg*‐silenced plants and TRV2:00 plants (Table S9, Supporting Information), where pathways linked to antioxidant activity (“superoxide dismutase activity”, GO:0004784, and “removal of superoxide radicals”, GO:0019430) were enriched among genes down‐regulated in *Ghfg*‐silenced plants (Figure S12C, Supporting Information). Heat maps of these genes illustrated the significantly different expression levels in TM‐1 and T582 bracts and the cell groups where genes were dominantly expressed (Figure 6B). We selected several genes involved in in auxin and antioxidant pathways for quantitative verification of their specific expression; the results were consistent with the enrichment analysis, thereby confirming the reliability of the RNA‐seq data (Figure 6C,D, Supporting Information). View together, the findings from the cell population enrichment and expression analysis indicate the alteration in bract morphology to be potentially linked with auxin homeostasis alongside oxidation and antioxidant processes.

### 2.6 Cells in Frego Bracts have Stronger Active Oxygen Scavenging Ability

**Figure 7:**
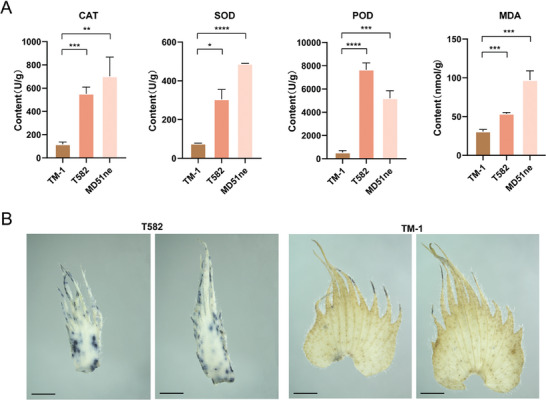
The ROS scavenging activity and distribution of two types bracts. A) The contents of catalase, superoxide dismutase, peroxidase, and the degree of membrane lipid oxidation in the bracts of three cotton varieties (*n* = 3 biological replicates). Student's *t*‐test and significance is denoted as *p < 0.05,**p < 0.01,***p < 0.001, ****p < 0.0001. Data are presented as mean ± s.d. B) The distribution of ROS in the two types of bracts was identified by NBT staining (*n* > 10 each group), bar = 1 mm.

Peroxidase content is a key indicator used to measure the antioxidant capacity of cells, and can also indicate the ability of plants to respond to stress conditions. We measured catalase (CAT), peroxidase (POD), and superoxide dismutase (SOD) contents in the young bracts of TM‐1 and T582, which revealed significant differences: frego bracts showed high levels of CAT, POD, and SOD, while in normal bracts, enzyme levels were relatively low (**Figure** **7**A). Additionally, nitrotetrazolium blue chloride staining and MDA content measurement indicated frego bracts to have a much higher degree of oxidation than normal bracts (Figure 7A,B), supporting the findings of our transcriptome analysis and suggesting *Ghfg* to have a role as a disease resistance gene.

### 2.7 Differential Phytohormone Distribution Determines Bract Morphology

As described above, analysis of single‐cell data revealed specific expression of auxin‐related genes in TM‐1 bracts. Accordingly, we measured phytohormone contents in normal (TM‐1) and frego (T582, MD51ne) bracts at multiple growth stages. All bracts were examined before bloom and were categorized based on length (Figure S13, Supporting Information) as either small (1–2 cm) or big (2–4 cm). Small bracts were divided into upside and downside sections, while big bracts were divided into upper side, middle side, and lower side sections. This segmentation strategy was adapted from the literature,^[^ ^35^, ^36^ ^]^ and allowed for a comprehensive analysis of hormone distribution. High‐performance liquid chromatography (HPLC) and mass spectrometry serial testing were used to quantify the contents of 3‐indoleacetic acid (IAA), cytokinin (ZR: CZR, TZR), and gibberellins (GAs: GA1/3/4/7), the detailed results of which are presented in Table S10 (Supporting Information).

**Figure 8:**
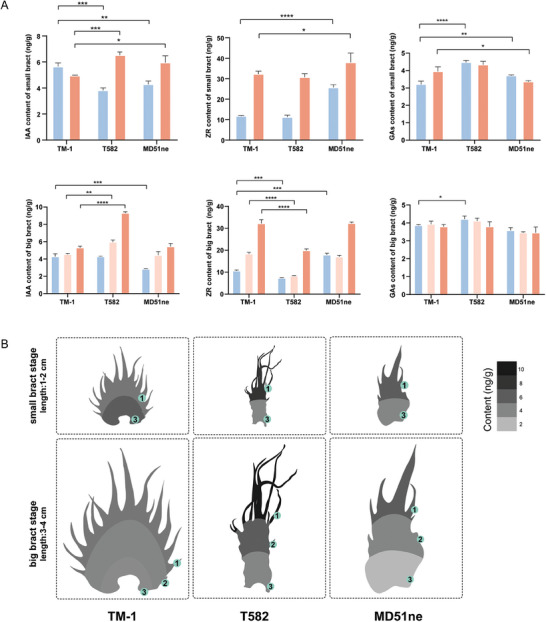
Phytohormone distribution of two types bracts A) The contents of IAA, ZR, GAs in different development stages and parts of bracts. Blue bars represent the part of “1”, orange bars represent the part of “3”, light pink bars represent the part of “2” (*n* = 3 biological replicates). Student's *t*‐test and significance is denoted as *p < 0.05,**p < 0.01,***p < 0.001, ****p < 0.0001. Data are presented as mean ± s.d. B) Bract distribution maps of IAA contents. The numbers in the green circles indicate the corresponding parts of the bracts (Figure S13, Supporting Information), the scale bar indicates the content values.

This analysis revealed clear differences in bract phytohormones across different periods, positions, and plant varieties. As expected, the distribution of auxin was notably distinct (**Figure** **8**A). For a more intuitive visualization, we plotted the distribution of auxin contents along the bract (Figure 8B). In the small bract stage, the contents difference between the upside and downside was smaller in TM‐1 bracts than in T582 and MD51ne, and the content within each section of the TM‐1 bracts was also significantly different from that in corresponding sections of T582 and MD51ne bracts.

At the big bract stage, TM‐1 bracts showed a relatively uniform IAA content across all three sections, with a slight increasing gradient from the base to the margin. The distribution of auxin in the bract margin was higher than that in the bract base could be linked to the development of leaf serrations, which is facilitated by the transport of auxin from the base to the margin through veins during leaf development. In contrast, the three sections of big T582 bracts displayed significant content discrepancies: the lower side resembled TM‐1 bracts in content, but the upper side was ≈9 ng g^−1^ higher, resulting in T582 having a more pronounced content gradient compared to TM‐1. In big MD51ne bracts, the IAA content gradient was not as pronounced, but was still noticeable; this likely contributes to the more evident longitudinal growth of MD51ne distal bracts. In terms of other hormones, such as ZRs, there were no significant differences in content distribution among the three varieties, nor between the two stages of bracts. A slight intervarietal difference in GA distribution was observed at the small bract stage, but this difference was eliminated by the big bract stage. View together, our results suggest that a uniform distribution of auxin is essential for the formation of fully formed heart‐shaped bracts, and the difference in the distribution of auxin between the proximal and distal ends contributes to the distinct morphology of *fg* bracts.

### 2.8 The Innovative Application of the Frego Bract in Sea‐Island Cotton

**Figure 9:**
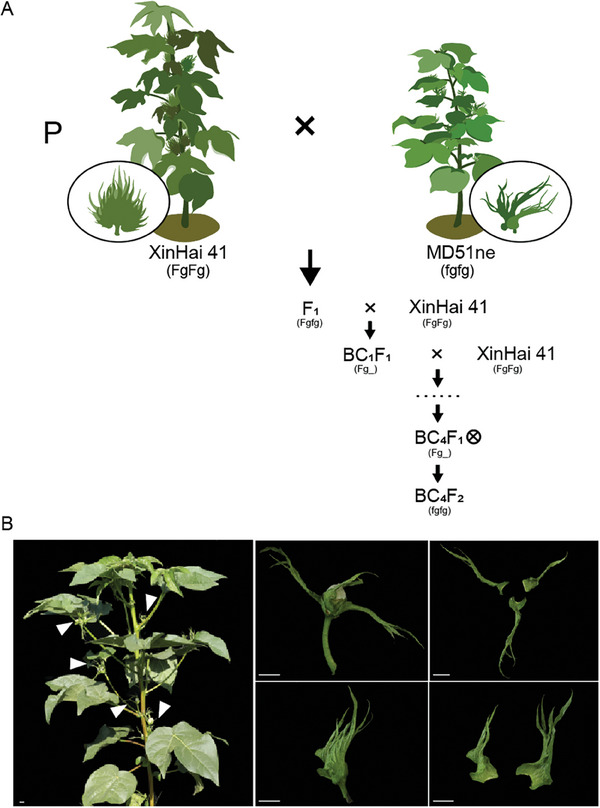
Breeding process and phenotype of Gb.CB. A) Breeding process of Gb.CB. FgFg, Fgfg and fgfg respectively represent the genotypes of normal bract, normal bract, and frego bract, Fg_ represents the genotypes of FgFg and Fgfg. B) Phenotype of Gb.CB and its’ bracts, bar = 10 mm.

Sea‐island cotton (*Gossypium barbadense*) exhibits superior comprehensive resistance and fiber properties compared to upland cotton (*Gossypium hirsutum*)^[^ ^37^ ^]^ making it highly valuable in commercial applications. However, sea‐island cotton also features larger bracts than upland cotton, and the presence of withered bracts in machine‐picked cotton fiber significantly diminishes its value. To address this issue, we introduced *Ghfg* into sea‐island cotton (**Figure** **9**A). Through a series of backcrosses involving F_1_ generations of Xinhai 41 and MD51ne with Xinhai 41 followed by repeated breeding up to the BC_4_F_1_ generation, self‐crossing of the BC_4_F_1_ population, and screening using the fg‐3 SNP marker (designed for KASP assays), we successfully obtained homozygous island cotton with the frego bract trait, named ″*G. barbadense* with curling bract, Gb.CB (Figure 9B). Gb.CB represents the first island cotton variety to incorporate the frego bract gene, and this innovative trait introduction is expected to enhance insect resistance and reduce the impurity content of machine‐picked island cotton fiber; it therefore, constitutes a significant advancement in breeding practice utilizing the *fg* gene.

## 3 Discussion

Cotton with the frego bract phenotype has exposed bolls, which is an important trait for pest resistance.^[^ ^12^, ^38^, ^39^ ^]^ Moreover, normal bracts often adhere to fibers during machine picking, while frego bracts are less attached and therefore produce lower impurity content in ginned cotton. Our previous work also revealed another advantage of frego bract, as cotton with this trait was found to be more convenient for hand‐selfing. Bracts also play a role in photosynthesis; the reduced area of frego bracts inevitably impacts their contribution to overall plant photosynthesis. Studies have shown that bracts contribute ≈25% of the photosynthetic intensity.^[^ ^6^ ^]^ The decrease in bract area in *fg* mutants therefore lowers the plant's photosynthetic intensity, leading to reduced dry matter accumulation and potentially explaining the smaller seed size of T582. Interestingly, while this reduction of photosynthesis capability might be expected to negatively impact cotton yield, many experiments have demonstrated the effect not significant.^[^ ^39^ ^]^ Percival and Kohel reported that the frego bract had no obvious effect on seed cotton yield, lint yield, and seed index.^[^ ^40^ ^]^ Specifically, the seed cotton yield of frego bract mutations are between 1881 and 1900 kg acre^−1^ while TM‐1 was between 1887 and 1922  kg acre^−1^. It indicated that although the boll size of frego bract cotton is small and the ginning outturn is low, this is compensated by the higher number of bolls per plant, resulting in frego bract cotton having similar seed and lint yields as TM‐1.

**Figure 10:**
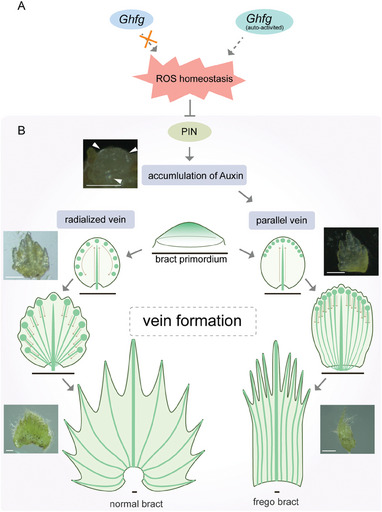
Model of two types veins of normal bract and frego bract formation. A) The *Ghfg* gene under the states of self‐activation would interfere with ROS homeostasis, while the self‐inhibited *Ghfg* would not. And ROS homeostasis would influence the auxin transfer by PIN. B) Both types of bracts first form a smooth bract primordium, along with the flow of auxin at the margins of bracts, PIN convergence points are formed which have the highest auxin concentration. The highest auxin concentration is equivalent to a new primordium from which a new vein is formed. Brown arrows represent the direction of auxin flow, green points represent the PIN convergence points, green lines represent the veins.

Although the cotton bract is a specialized and modified leaf, the mechanisms regulating its development remain understudied. The successful cloning of the *fg* gene in this work could enhance our comprehension of the regulatory network that underlies bract development. Through our transcriptomic analysis of *Ghfg* VIGS plants, we observed down‐regulation of several genes associated with ROS clearance. Intriguingly, we also identified genes specifically expressed in the proliferating cells of T582 bracts that were associated with SOD activity and NADP/NAD binding. These findings suggest that SOD activity in frego bracts surpasses that in normal bracts. *Ghfg* as a class of immune‐related proteins triggers downstream immune responses such as activating SOD or peroxidase to either induce or eliminate ROS, and thereby affects ROS homeostasis. We propose that a point mutation in *Ghfg* leads to its self‐activation, which results in ROS accumulation that alters auxin distribution and transport. It is established that ROS can regulate the polar transport of auxin by affecting both the efflux and influx‐dependent transport of auxin.^[^ ^41^ ^]^ *Arabidopsis* plants with abnormal ROS levels exhibit impaired polar transport of auxin, leading to transient inhibition of auxin signaling in leaves and roots.^[^ ^42^, ^43^ ^]^ Elevated ROS level also disrupts the endogenous auxin distribution in the root apical meristem, causing reduced proliferation and retarded growth of the primary root.^[^ ^44^ ^]^ When we analyzed the RNA‐seq data of TM‐1 and T582 bracts (Figure S14, Supporting Information), we found that some genes related to auxin efflux and auxin polar transport were highly expressed in TM‐1 bract, such as PINFORMED1 (PIN1) and PINFORMED3 (PIN3). The same trend was observed after VIGS silencing *Ghfg*, however, the expression of genes related to the clearance of ROS was downregulated. This means that genes regulating auxin transport and ROS homeostasis are downstream of *Ghfg*, and silencing *Ghfg* would affect the expression of these genes. Combining these findings with our studies, we developed a model illustrating how *Ghfg* regulates PIN transport of auxin by modulating ROS homeostasis (**Figure** **10**A).

Auxin plays an important role in leaf polarity establishment, and its polarity transport is crucial for determining the final leaf shape.^[^ ^44^ ^]^ We found genes up‐regulated in normal bracts (TM‐1) to be enriched for terms related to auxin activity and auxin transportation, supporting our initial hypothesis. Auxin is synthesized in vascular and epidermal cells,^[^ ^45^ ^]^ then is transported from the vascular bundle to the distal end of the leaf, whence it flows to the margins. In this process, auxin maxima termed PIN1 convergent points are formed through the interplay between auxin and PIN1 protein.^[^ ^46^ ^]^ These convergent points give rise to new primordia that mark the end points of newly formed veins at which serration and cleft formation are subsequently initiated, thus shaping the leaf.^[^ ^47^ ^]^

Our analysis of phytohormone distributions revealed auxin to be evenly distributed in normal bracts, but to accumulate at the tip in frego bracts, producing a significant content gradient between base and tip. This suggests that in normal bracts, auxin flows from the bract tip to the margins, gradually forming evenly distributed convergent points, serrations, and new veins.^[^ ^48^ ^]^ This process is consistent with canalization theory.^[^ ^49^ ^]^ and with observations in maize, where regulation of medial‐lateral growth and vein formation depends on mutual regulation of auxin with cytokinin and gibberellic acid, and on sustained auxin response maxima during continuous growth in the proximal margins.^[^ ^50^ ^]^ Meanwhile, in frego bracts, auxin accumulates at the distal end, convergent points are concentrated in the upper part of the bract. As veins differentiate along the path of auxin flow, this results in the formation of parallel veins (Figure 10B). As the veins drive leaf development and provide the motive force for leaf expansion,^[^ ^46^ ^]^ this ultimately leads to the formation of long and narrow bracts. These differences in the overall distribution of auxin were also reflected in cell morphology. In our observation of surface cells, the epidermal cells of frego bracts were elongated while those of normal bracts extended in all directions, with more uniform cell morphology and growth. We searched for some genes related to leaf development in RNA‐seq data, such as *KANAD* (*KAN 1/2*), *YABBY* (*YABs*)^[^ ^51^, ^52^ ^]^ and *WUSCHEL‐RELATED HOMEOBOX 3* (*WOX 1/3*)^[^ ^53^ ^]^ these genes regulate the expression of auxin transport genes and cell polarity differentiation, as well as genes related to leaf margin development, such as *JAGGED* (*JAG*) *PETAL LOSS* (*PTL*)^[^ ^54^, ^55^ ^]^ *ASYMMETRIC LEAVES* (*AS1/2*)^[^ ^52^ ^]^ all of them were highly expressed in TM‐1 and it confirms our cytological observations (Figure S15, Supporting Information). This also means the polar transport direction of auxin was changed, resulting in medial‐lateral growth being slower than proximal‐distal growth, producing the narrow and curling bracts of *fg* mutant plants.

## 4 Conclusion

Our research on cotton bracts also serves as a reference for other plants with bracts. The study of bracts as a leaf type is relatively lacking, and the functions of many mutants have not been explored and applied. Developing a better understanding of bracts and their morphological characteristics is of great significance not only for the determination of gene functions but also to better apply that knowledge to flower organ protection and production applications.

## 5 Experimental Section

### Plant Materials

*G. hirsutum* acc. TM‐1 (Texas Marker‐1) is a standard genetic line^[^ ^22^ ^]^ Texas 582 (T582) is a multiple‐recessive marker line with the same genetic background as TM‐1^[^ ^21^ ^]^ *G. hirsutum* acc. MD51ne^[^ ^56^ ^]^ is a multispecies hybrid (*G. tomentosum* Nattall ex. Seemann, *G. arboreum* Linn., *G. thurberi*, *G. hirsutum*, *G. barbadense*) that is nectaryless, has high fiber strength,^[^ ^57^ ^]^ and has frego bracts (Figure S1C, Supporting Information). In 2015, TM‐1 and T582 were crossed at the Jiangpu Breeding Station of Nanjing Agricultural University in China for the development of an F_2_ mapping population (2164 individuals) and a BC_1_ population (412 individuals). *Nicotiana benthamiana* was used for the instantaneous conversion assay.

### Map‐Based Cloning of the Ghfg Gene

We extracted DNA by means of the CTAB method^[^ ^58^ ^]^ from 28 frego bract individuals out of BC_1_ (TM‐1×T582) population progeny. DNA was quantified using a Nanodrop 2000 spectrophotometer (Thermo Fisher, United States). DNA was bulked in equal proportions to generate a mutant‐type pool, and then whole‐genome resequencing conducted with T582 on the Illumina HiSeq 2500 platform using 2 × 101 bp reads. Reads were trimmed with Sickle and aligned to the TM‐1 reference genome (TM‐1_v1.1)^[^ ^23^ ^]^ using BWA.^[^ ^59^ ^]^ Sorting and subsequent SNP and InDel identification were performed by SAMtools,^[^ ^60^ ^]^ and candidate regions were identified according to the difference ratio of SNPs in sequenced individuals.^[^ ^15^ ^]^ Molecular markers designed with the PRIMER 3.0 software (Table S1, Supporting Information) were then used to further localize *Ghfg* to a 190.4 kb region. We then mapped this interval to the TM‐1_v2.1 genome,^[^ ^24^ ^]^ which yielded a 1 Mb candidate interval.

### Kompetitive Allele Specific PCR (KASP) Assay

To verify the accuracy of the mapping and to further find key variants in the mapping interval, KASP markers were selected from SNPs confirmed by second‐generation sequencing. KASP primers (Table S1, Supporting Information) were designed by software offered by LGC Genomic (LGC, Hoddesdon, UK), and the KASP assay was performed in a 1.6 µL PCR reaction system including 0.8 µL (15–30 ng) of template DNA, 0.8 µL of 2× KASP master mix (LGC, Biosearch Technologies), and 0.07 µL of primer. PCR was performed following the assay manufacturer's instructions (LGC, Biosearch Technologies) using an IntelliQube (LGC, Biosearch Technologies). After reading and analyzing the fluorescence data, the results were visualized in the form of genotyping maps (Table S3, Supporting Information).

### Quantitative RT‐PCR Analysis

Total RNA was extracted from cotton bracts using the Plant RNA Rapid Extraction Kit (Molfarming, Nanjing, China). First‐strand cDNA was generated using the HiScript II QRT SuperMix for qPCR (+gDNA wiper) kit (Vazyme, Nanjing, China). The cotton ubiquitin gene (GenBank accession no. AY189972) was used as the reference gene, and the sequences of the qRT‐PCR primers are listed in Table S1 (Supporting Information). Three biological replicates were performed per reaction, each with three technical replicates. Mean values and standard errors were calculated based on data from three replicates. ChamQ Universal SYBR qPCR Master Mix (Vazyme, Nanjing, China) was used in the expression analysis of target genes, for which PCR conditions were according to the product instructions.

### Virus‐Induced Gene Silencing (VIGS) Assay

As *Ghfg* has a long coding sequence, three silencing fragments of 201 bp each were utilized in order to achieve a complete silencing effect. The fragments were amplified from the cDNA of T582 and were cloned into the pTRV2 vector to produce the construct set referred to as pTRV2:*Ghfg* (TRV::*Ghfg‐1/2/3*). The recombination primers are listed in Table S1 (Supporting Information). Silencing (pTRV2:*Ghfg*), viral function (pTRV1), and control constructs (pTRV2:*CLA*, positive control; pTRV2, negative control)^[^ ^61^ ^]^ were transfected into *Agrobacterium tumefaciens* strain GV3101, cultured overnight, centrifuged, and adjusted to a OD 600 of 1.5–2.0 with infiltration medium (10 mm MgCl_2_, 10 mm 2‐(*N*‐morpholino) ethanesulfonic acid [MES], and 200 mm acetosyringone)^[^ ^57^, ^58^ ^]^ *Agrobacterium* strains containing the pTRV1 and pTRV2 vectors were mixed at a ratio of 1:1 and then injected into the cotyledons of thirty 10‐day‐old T582 seedlings with 1 mL injection syringes,^[^ ^62^, ^63^ ^]^ after which the plants were placed in the dark for 24 h, then incubated at 23** °**C with a 16‐h light/8‐h dark cycle. Photos and samples were taken 45 days after injection while the bracts were fully unfolded.

### RNA In Situ Hybridization

Fresh bract samples of TM‐1 were collected at about seven days after squaring (bract length ≈5 mm) and embedded in paraffin. mRNA was detected using the MK2048 FGF8 mRNA in situ hybridization kit (BOSTER, Wuhan, China). The sequences of the probes used are listed in Table S1 (Supporting Information).

### Protoplast Isolation and scRNA‐Seq Library Construction

Fresh bract samples of T582 and TM‐1 were collected at about seven days after squaring (bract length ≈5 mm). After degerming, the bract was cut into 1–2 mm strips, placed into tubes, enzyme solution added (1% cellulase R‐10, 0.2% macerozyme R‐10, 0.3% pectinase Y‐23, 0.1% BSA, 20 mm MES, 20 mm KCl, and 10 mm CaCl_2_, pH 5.5–5.8), and then stood or slightly shook at 28 °C for 1.5–3 h in the dark. The reaction was terminated when the cells were visible under a microscope. Cell activity was detected by trypan blue staining and cell concentration was measured using a hemocytometer and a light microscope, then adjusted to 400–1200 cells µL^−1^. Protoplasts were loaded onto the 10× Genomics Chromium platform and wrapped in oil droplets to formed a single‐cell microreactive system. After the Gel Beads in Emulsion (GEMs) were formed, they were collected and reversed for labeling by PCR. The GEMs were then treated with an advanced oil break and the cDNA purified and enriched with beads to construct the library. Last, the library was sequenced on an Illumina NovaSeq 6000 sequencer (Illumina).

### ScRNA‐Seq Data Processing

The reference genome (TM‐1_v2.1) and annotation files for *G. hirsutum* were obtained from COTTONOMICS (http://cotton.zju.edu.cn/). The Cell Ranger (v.7.1.0) software was used to perform the alignment of raw sequencing data (Figure S5, Supporting Information). Seurat^[^ ^64^ ^]^ and DoubletFinder^[^ ^65^ ^]^ were used for quality control to remove substandard data and outlier values such as double cells, dead cells, cell fragments, cells expressing too few or too many genes (≤ 300 or ≥ median ± 3 × median absolute deviation), and undesirable UMI numbers (UMI ≤ 500 or UMI ≥ 30000) (Figures S6–S8, Supporting Information); additionally, mitochondrial and ribosomal genes were omitted. We then used Harmony^[^ ^66^ ^]^ to combine TM‐1 and T582 data and perform batch correction. The single‐cell transcriptomes were then grouped into 12 distinct clusters (Table S4, Supporting Information). We used the expression of previously reported marker genes (Table S6, Supporting Information)^[^ ^28^, ^29^, ^30^, ^31^ ^]^ in clusters to further identify each cluster and carry out the next analysis.

### RNA‐Seq Data Processing

Bracts were collected from TM‐1, T582, TRV2:*Ghfg*, and TRV2:00 plants, with three biological replicates sampled for each tissue. Total RNA was extracted as mentioned above. After a quality inspection of the library, high‐throughput sequencing was performed using the Illumina sequencing platform. The quality of the resulting transcriptome data was assessed using FastQC (v0.11.8)^[^ ^67^ ^]^ All clean reads were aligned to the TM‐1 reference genome (TM‐1_v2.1) using Hisat2 (v2.2.0)^[^ ^68^ ^]^ and StringTie (v1.3.5) was used to quantitate the alignment results. FeatureCounts (v2.0.0) was used to obtain the raw input data for downstream recounts. Gene expression was expressed in terms of the transcripts per kilobase of the exon model per million mapped reads (TPM). Counts were entered as the original quantitative data and normalized using the rlog function. Analysis of differentially expressed genes (DEGs) was carried out using the R package *DESeq2* (v1.36.0)^[^ ^69^ ^]^ with absolute value of log2 (fold‐change) ≥ 1 and adjusted *P*‐value ≤ 0.05 as the screening criteria (Tables S2,S9, Supporting Information). Heatmaps were generated using the R package *pheatmap* (v1.0.12)^[^ ^70^ ^]^ and Venn diagrams using the R package *VennDiagram* (v1.6.0)^[^ ^71^ ^]^ Gene Ontology (GO) enrichment analysis used the database from COTTONOMICS (http://cotton.zju.edu.cn/), and plots were generated with the R packages *clusterProfiler* (v4.10.0)^[^ ^72^ ^]^ *stringr* (v1.5.1) (https://CRAN.R‐project.org/package=stringr) and *ggplot2* (v3.4.4) (https://CRAN.R‐project.org/package=ggplot2).

### Phytohormone Detection and Enzyme Activity Detection

The phytohormone content was determined by Nanjing Convinced‐test Technology Co., Ltd (Nanjing, China). The bracts were collected from TM‐1, T582, and MD51ne plants before blossoming and grouped according to length (small bracts: 1–2 cm, big bracts: 2–4 cm). Those in the small bract group were divided into two parts and those in the big bract group into three parts as illustrated in Figure S13 (Supporting Information). Three replicates were taken from each variety, and the samples were snap‐frozen in liquid nitrogen immediately.^[^ ^73^ ^]^ After grinding into dry powder in liquid nitrogen, an isopropyl alcohol–water–hydrochloric acid mixed extract and 8 µL (1 µg mL^−1^) internal standard solution (IAA standard: Dr.ehrenstorfer, Germany; CZR standard/GA(1/3/4/7): Olchemim, Shanghai, China; TZR standard: J&K, Beijing, China) was added to each sample. Dichloromethane was then added and a lower organic phase separated by centrifugation; this phase was dried by nitrogen, then redissolved in methanol (0.1% formic acid). After a final centrifugation, a portion (2 µL) of the solution was analyzed using an LC‐ESI‐MS/MS system consisting of an HPLC (Agilent 1260) system couple to Tandem mass spectrometry (MS/MS) (Applied Biosystems 6500 Quadrupole Trap). The software Analyst was used to process mass spectrum data. The contents of CAT, SOD, POD, and MDA were detected by an enzyme activity kit (Solarbio, Beijing, China). The bracts which before blossoming were quickly frozen with liquid nitrogen, ground into powder. The enzymes were extracted according to the kit instructions, and tested by spectrophotometer.

## Conflict of Interest

The authors declare no conflict of interest.

## Author Contributions

Conceptualization was done by T.Z.Z. and Y.H. Methodology was dealt by Y.H., Z.F.S., and Y.H.Z. Investigation was done by S.Y.Y., L.S.X., L.H., Y.W.C., and X.R.L. Visualization was done by G.A.Q. Supervision was done by T.Z.Z. and Y.H. Original draft was wrote by S.Y.Y. Y.H. wrote the review and editing. All authors discussed results and commented on the manuscript.

## Supporting information

Supporting Information

Supplementary Table S1

## Data Availability

The data that support the findings of this study are available from the corresponding author upon reasonable request.
