# Supplementary material for: A CC‐NB‐ARC‐LRR Gene Regulates Bract Morphology in Cotton
Source: Adv Sci (Weinh). 2024 Oct 4;11(44):2406111. doi: 10.1002/advs.202406111 (PMC11600217; doi:10.1002/advs.202406111)
Supplement: Supplementary file 1 — Supporting Information [file ADVS-11-2406111-s002.docx]

**Supplementary figures**


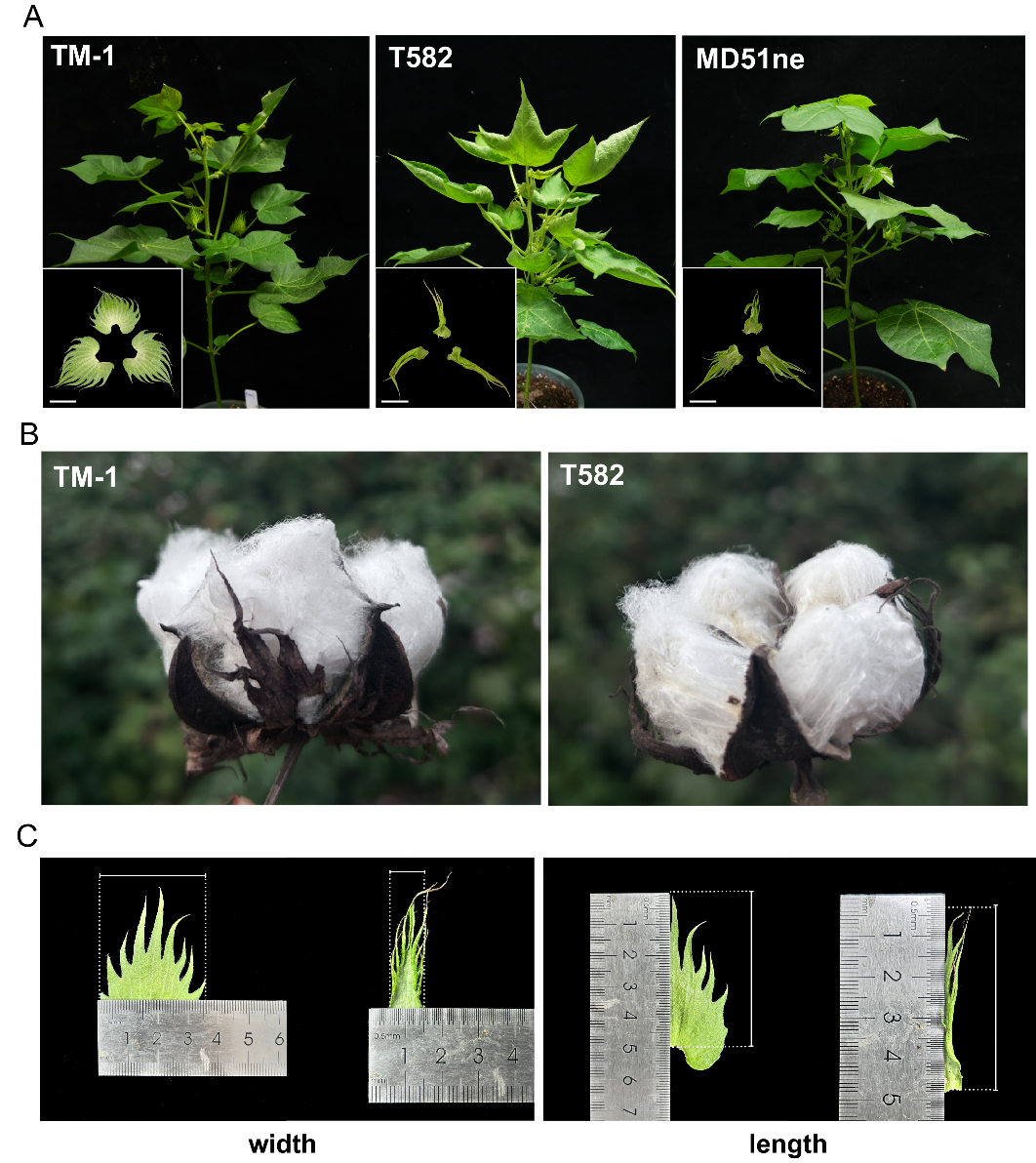


**Figure S1. Plant morphology of different cotton varieties.** (A) Plants of TM-1, T582, MD51ne and morphology of bracts. Bar = 10 mm. (B) Boll opening of TM-1 and T582. (C) Method for measuring the length and width of the bracts.


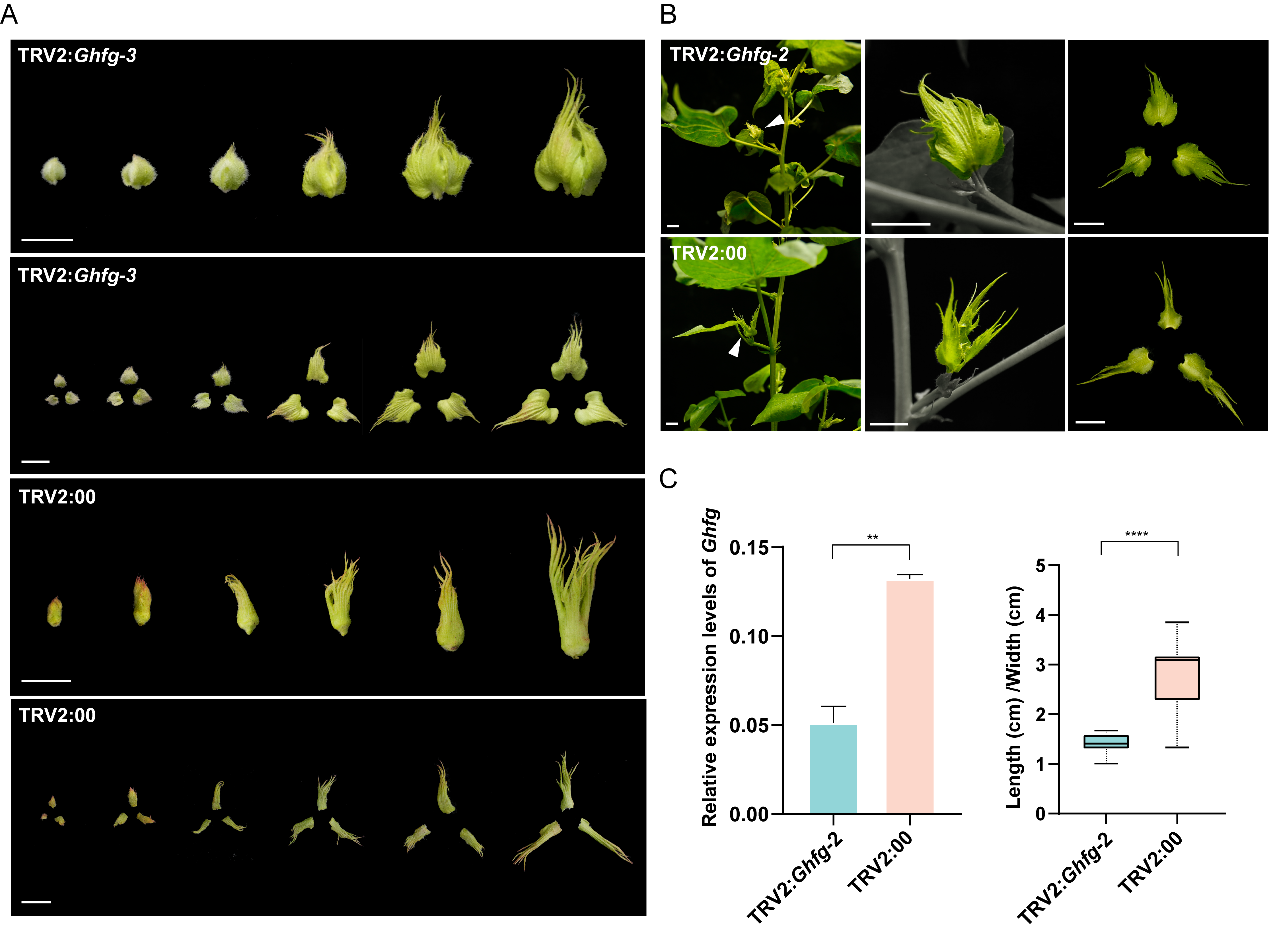


**Figure S2. The phenotypes after VIGS.** (A) After silencing *Ghfg-3*, the restoration process of T582 bracts at different growth stages compared with TRV2:00 group, bar = 10 mm. (B) Silencing *Ghfg-3* in MD51ne plants showed the changes of bracts. (C) qRT-PCR result of VIGS (n = 3 biological replicates) and the ratio of length/width of bracts (n = 18 each group), bar = 10 mm. Statistical analysis was performed using Student's *t*-test, and significance is denoted as ***p* < 0.01, *****p* < 0.0001. Data are presented as mean ± s.d.


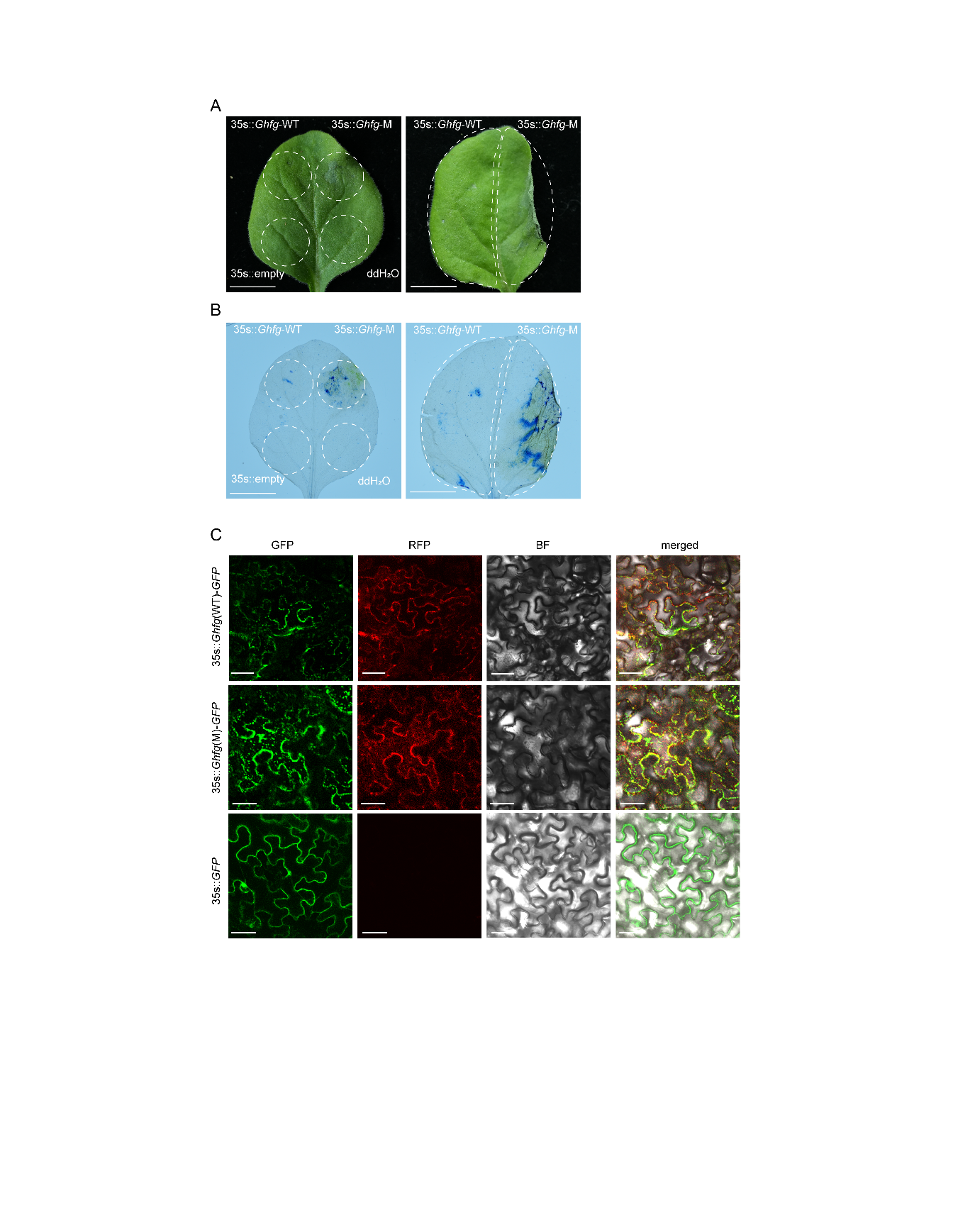


**Figure S3. *Ghfg* self-activation test after mutation.** (A) Tobacco leaf 72 hours after injection (n = 22 each group), bar = 10 mm. *Ghfg-*WT represents the gene before the mutation (pre-mutated), *Ghfg-*M represents the gene after the mutation (post-mutated), 35s::empty and ddH_2_O represent the vector control group and blank control group. (B) Tobacco leaf dyed with Trypan blue. Bar = 10 mm. (C) Subcellular localization of *Ghfg*-WT and *Ghfg*-M. Confocal images are from tobacco leaf epidermis co-expressing the GFP fusions and the marker *AtNIP1*. Bar = 50 μm.


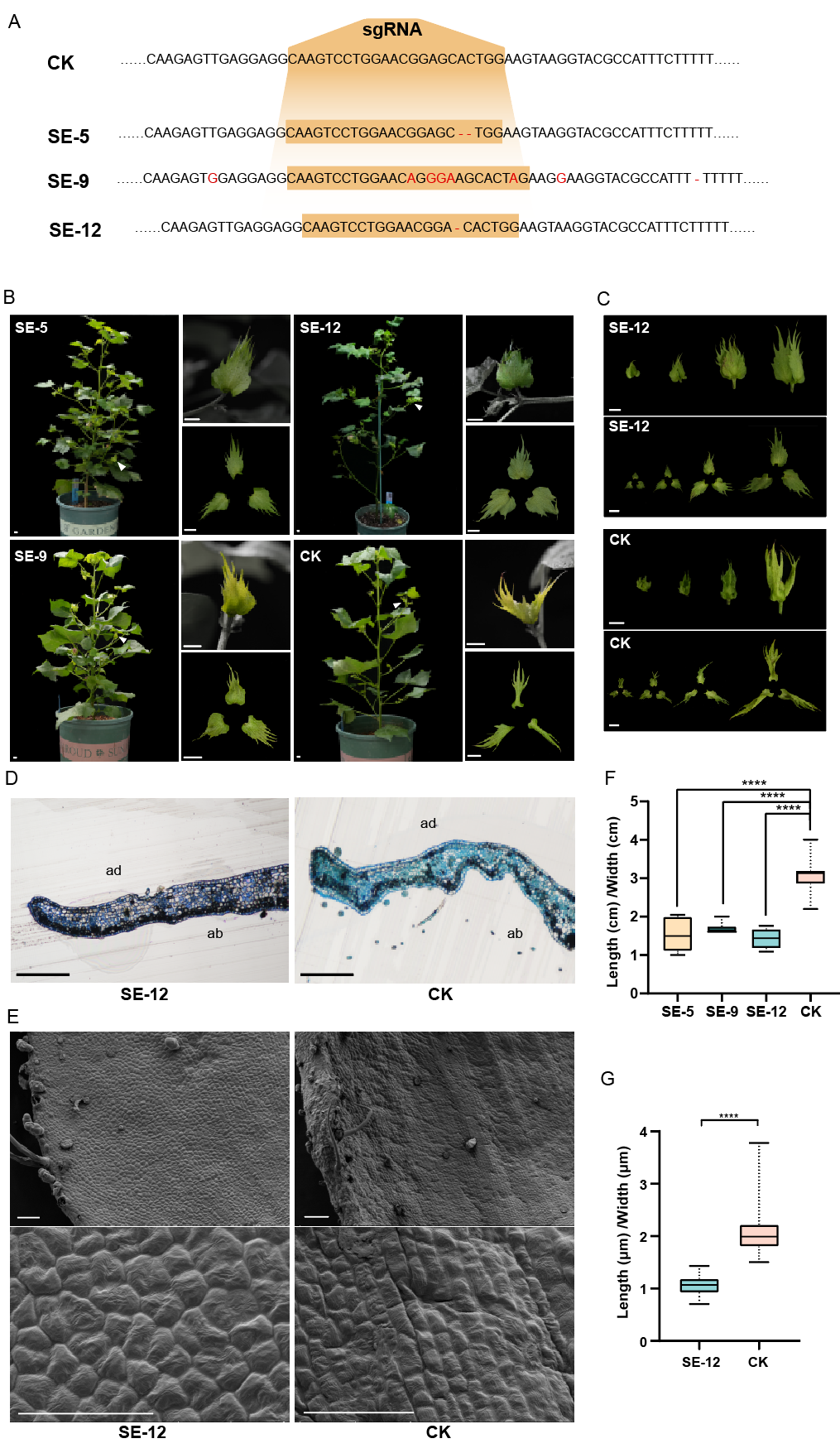


**Figure S4. Phenotypes of MD51ne after CRISPR/Cas9.** (A) The edit forms of three lines after CRISPR/Cas9. (B) The phenotypes of line SE-5, SE-9, SE-12, bar = 10 mm. (C) The restoration process of SE-12 bracts at different growth stages compared with CK, bar = 10 mm. (D) Reson sections of SE-12 and CK, bar = 100 μm, ad: adaxial, ab: abaxial. (E) Morphology of epidermal cells of SE-12 and CK, bar = 50 μm. (F) The ratio of length/width of transgenic lines and CK (n = 12 each group). (G) Length to width ratio of bracts cells of SE-12 and CK (n = 40 each group). Statistical analysis was performed using Student's *t*-test, and significance is denoted as *****p* < 0.0001. Data are presented as mean ± s.d.


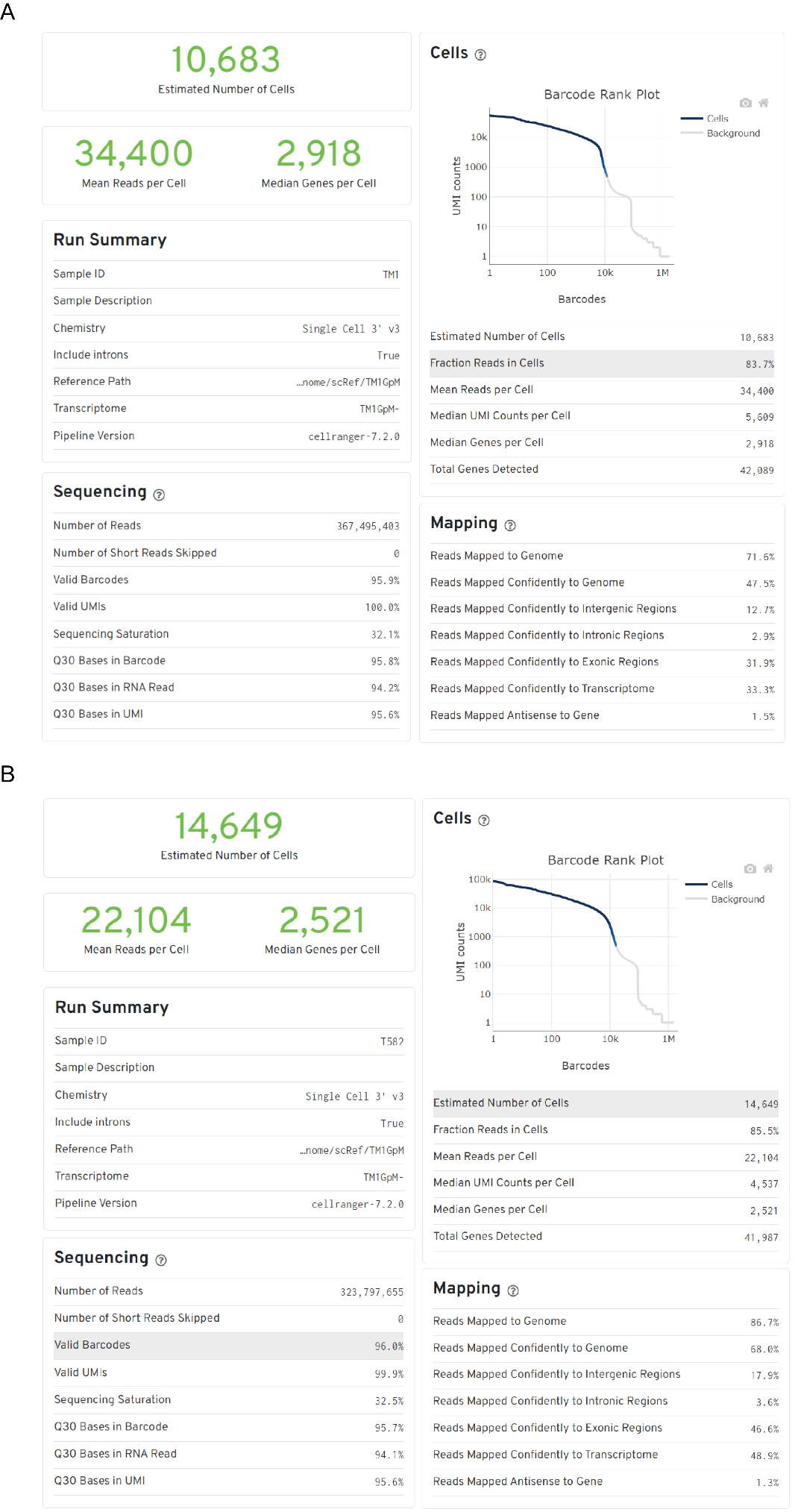


**Figure S5. Brief chats of Cell Range software report.** (A) TM-1 bract. (B) T582 bract.


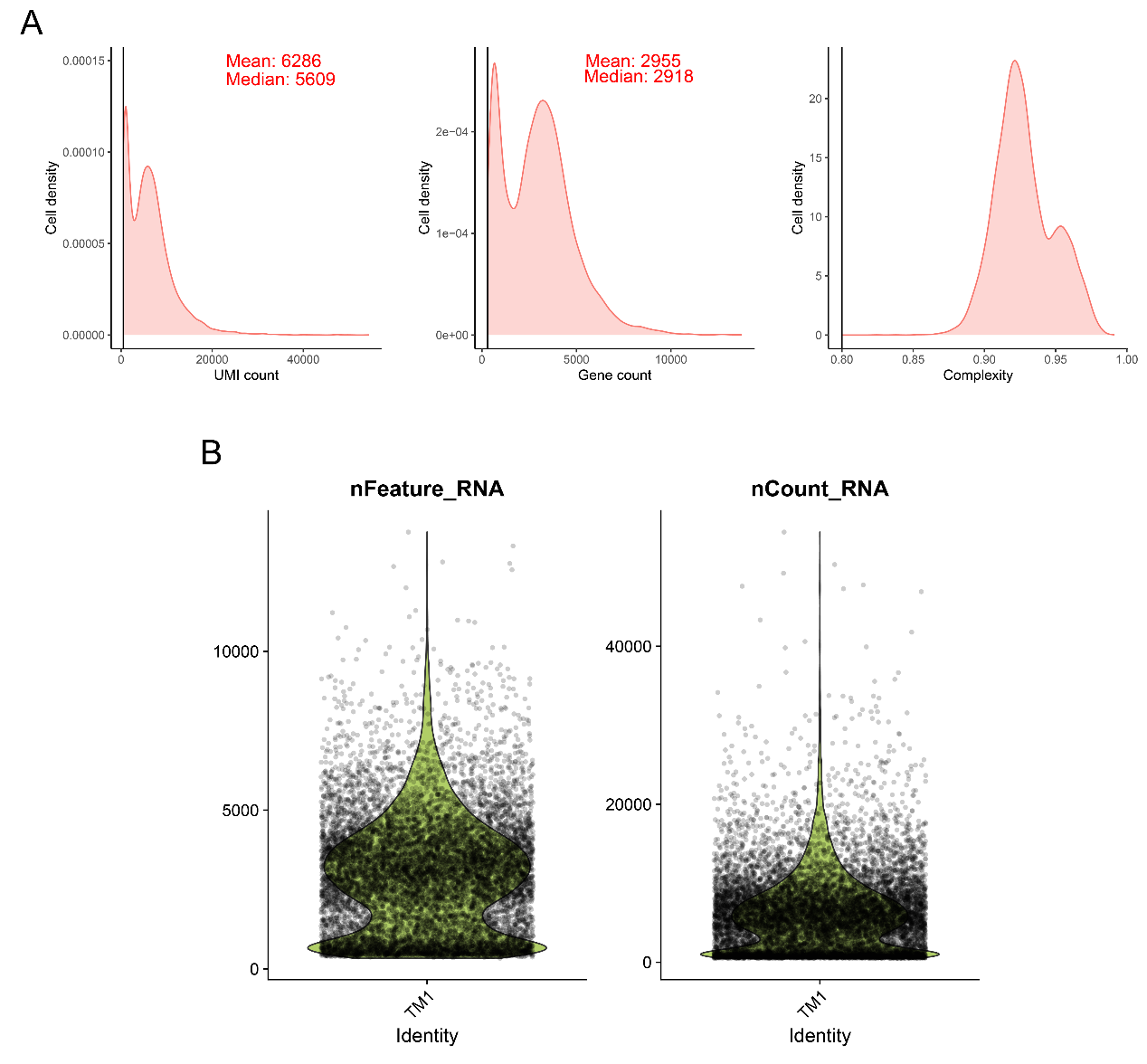


**Figure S6. TM-1 bract data quality control.** (A) Density curves of UMI and gene count. (B) Dot plots of nFeature_RNA and nCount_RNA.


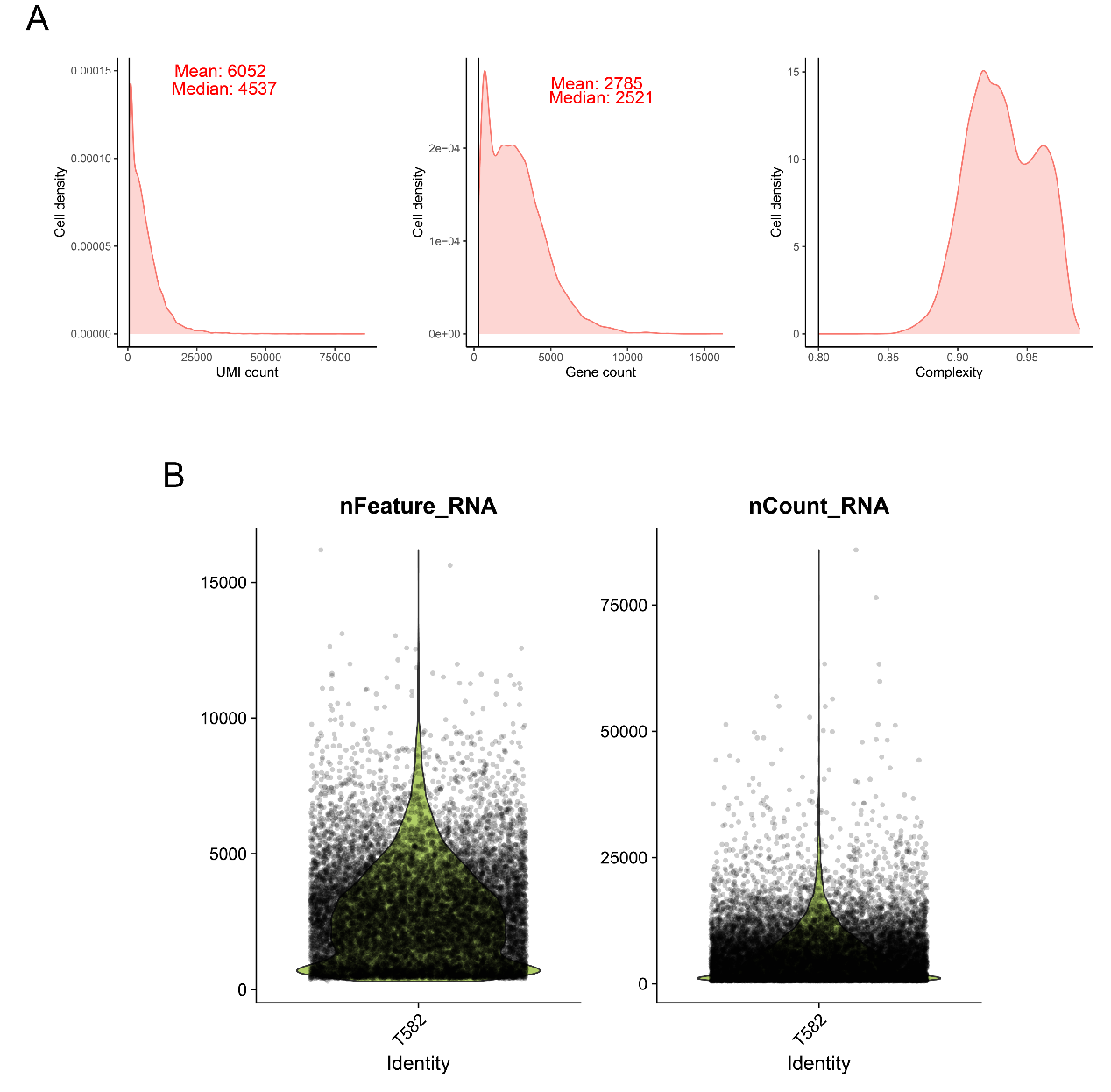


**Figure S7. T582 bract data quality control.** (A) Density curves of UMI and gene count. (B) Dot plots of nFeature_RNA and nCount_RNA.


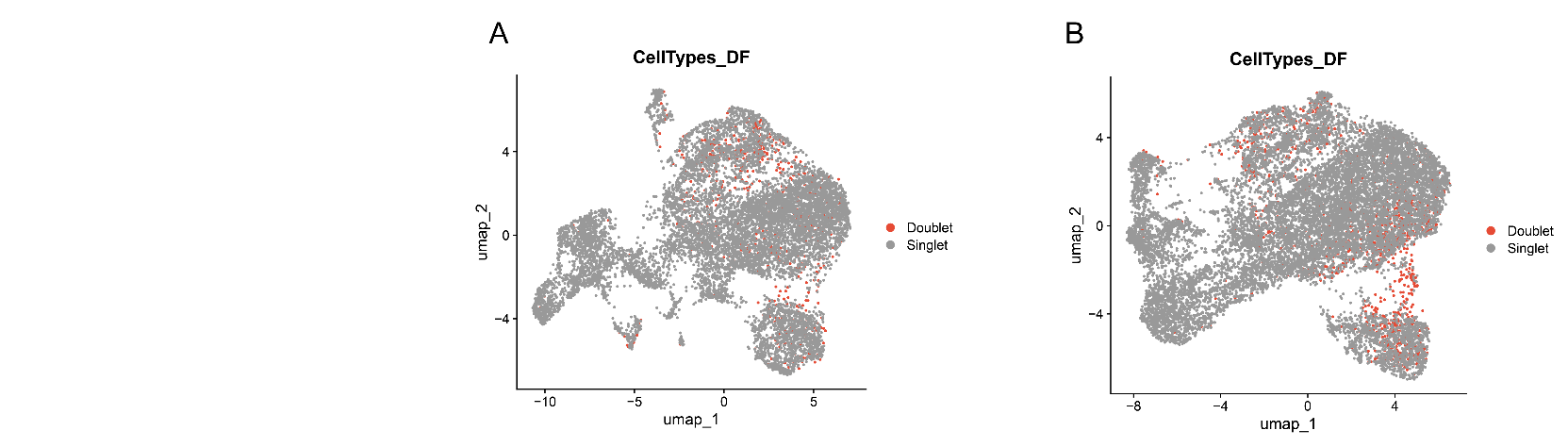


**Figure S8. Doublet removing.** (A) TM-1 bract. (B) T582 bract.


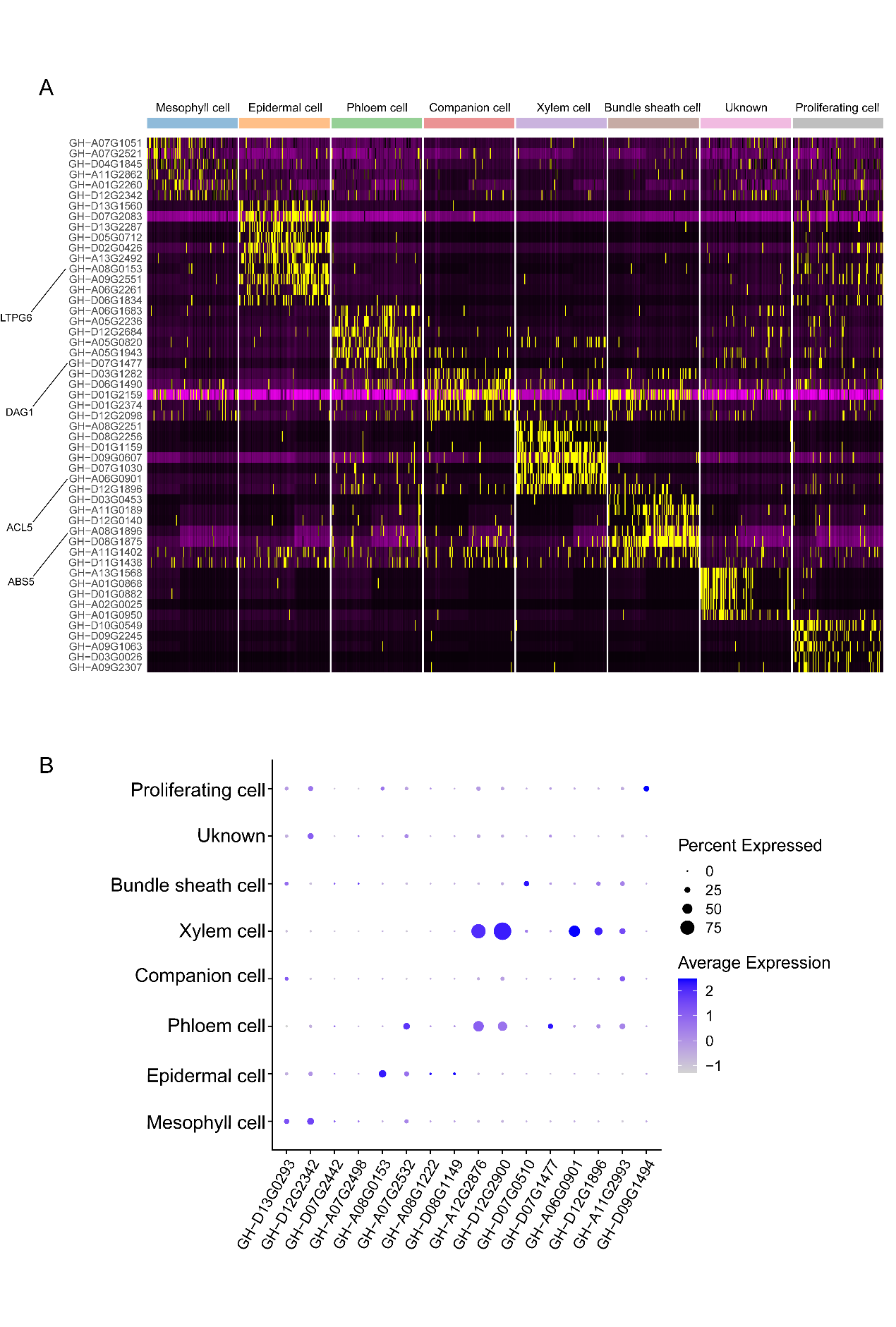


**Figure S9. Heatmap of the highest expression genes in each cluster and the dot plot of marker genes.** (A) Heatmap of the highest expression genes. (B) Dot plot of marker genes.


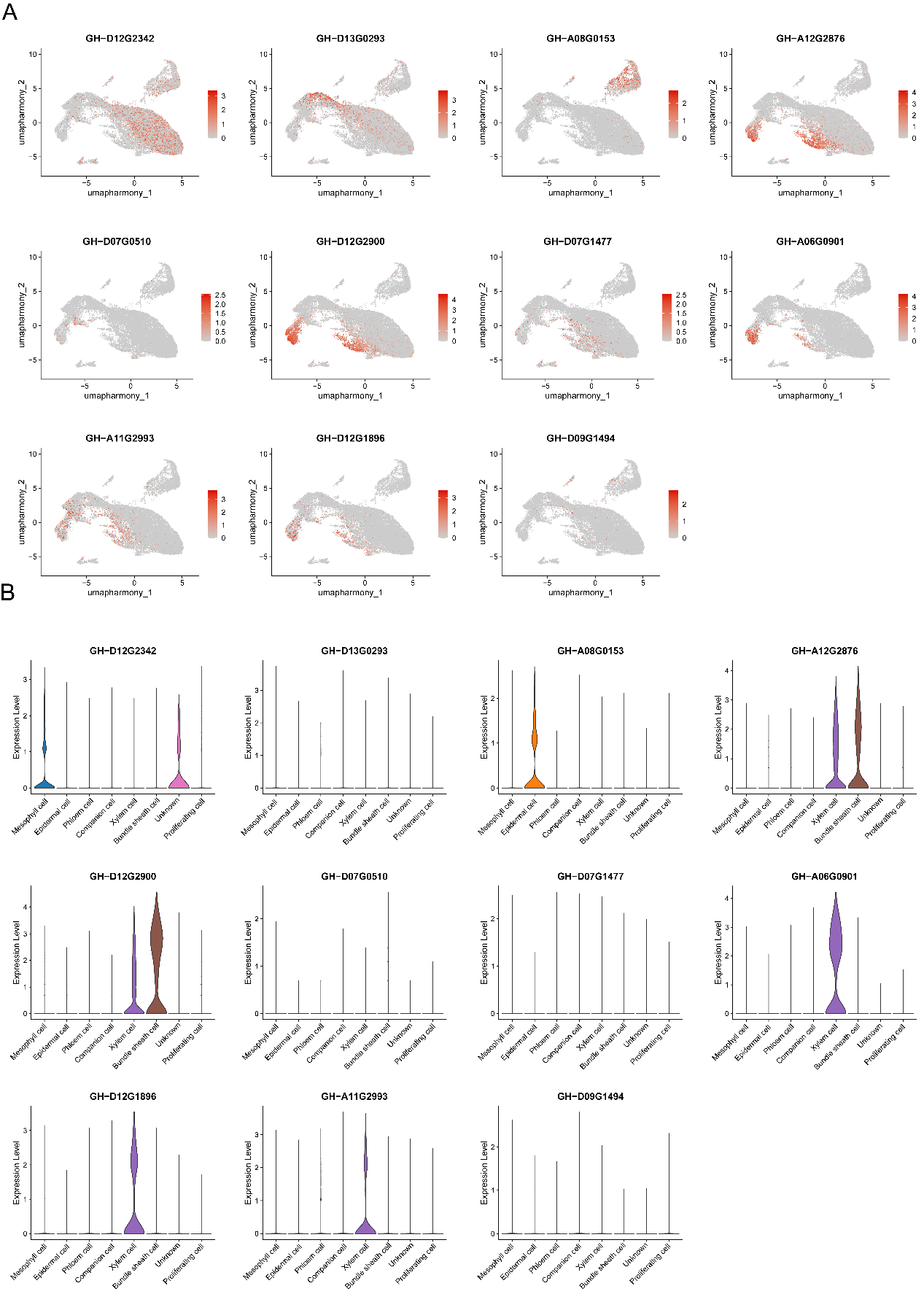


**Figure S10. Marker genes distribution in UMAP and violin plots.**


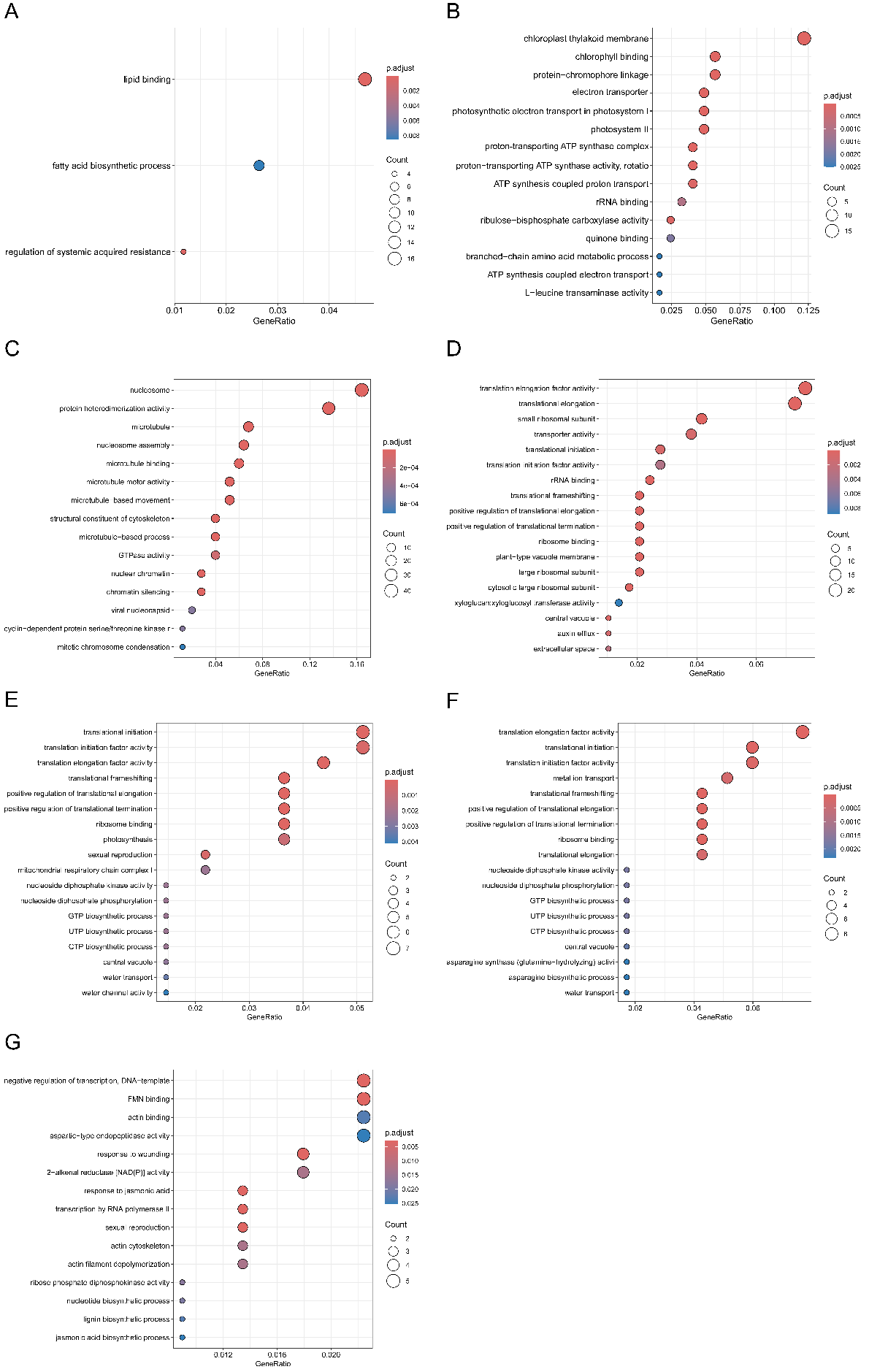


**Figure S11. GO enrichment of each cluster.** (A) epidermal cell. (B) mesophyll cell. (C) proliferating cell. (D) xylem cell. (E) bundle sheath cell. (F) companion cell. (G) phloem cell. The color of each dot represents the statistical significance (p. adjust) of the enriched terms, and the diameter of the dot indicates the number of genes associated with each term.


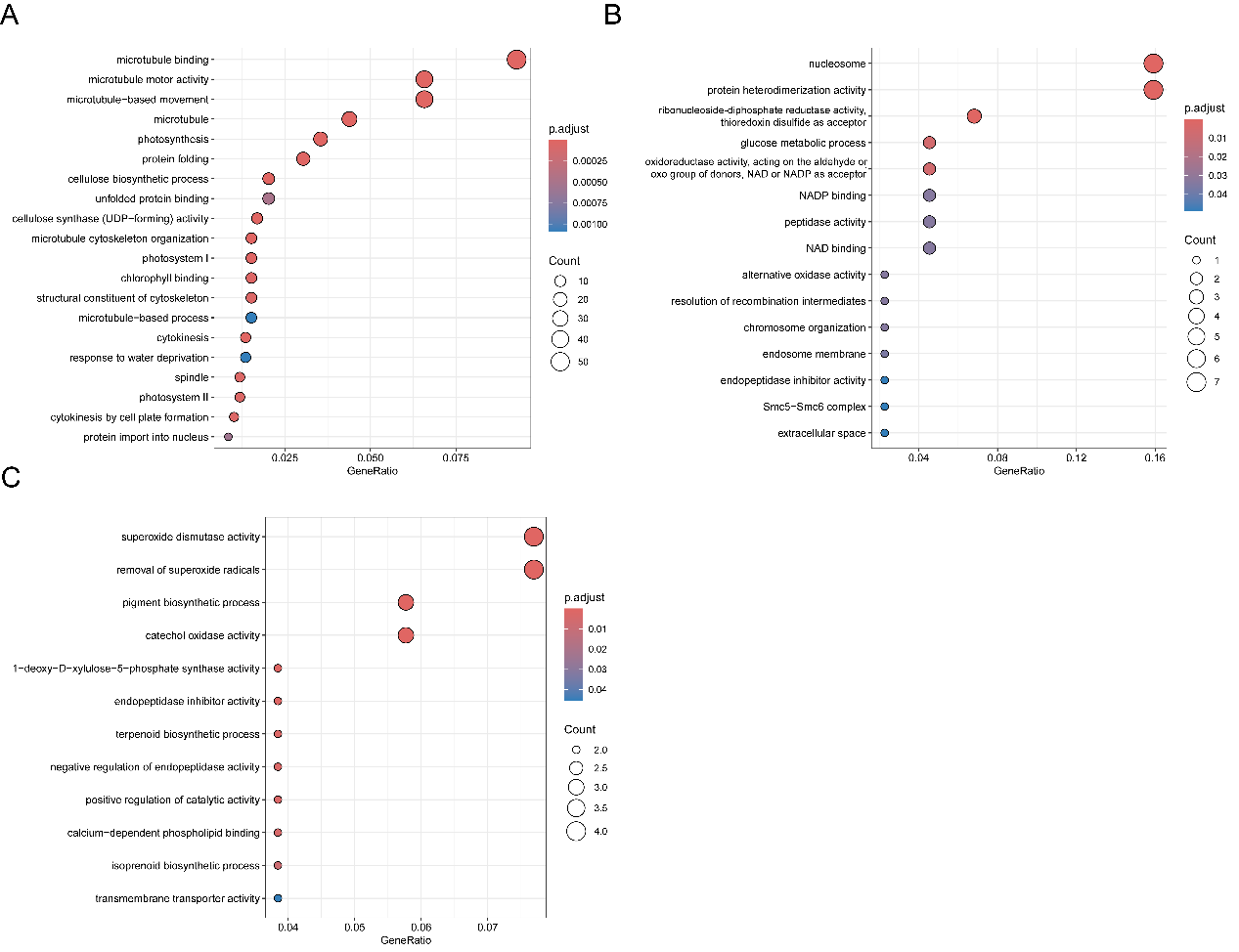


**Figure S12. GO enrichment.** (A) Proliferating cell of TM-1 bract. (B) Proliferating cell of T582 bract. (C) Downregulation genes in TRV2:*Ghfg* compared to TRV2:00 after VIGS in T582. The color of each dot represents the statistical significance (p. adjust) of the enriched terms, and the diameter of the dot indicates the number of genes associated with each term.


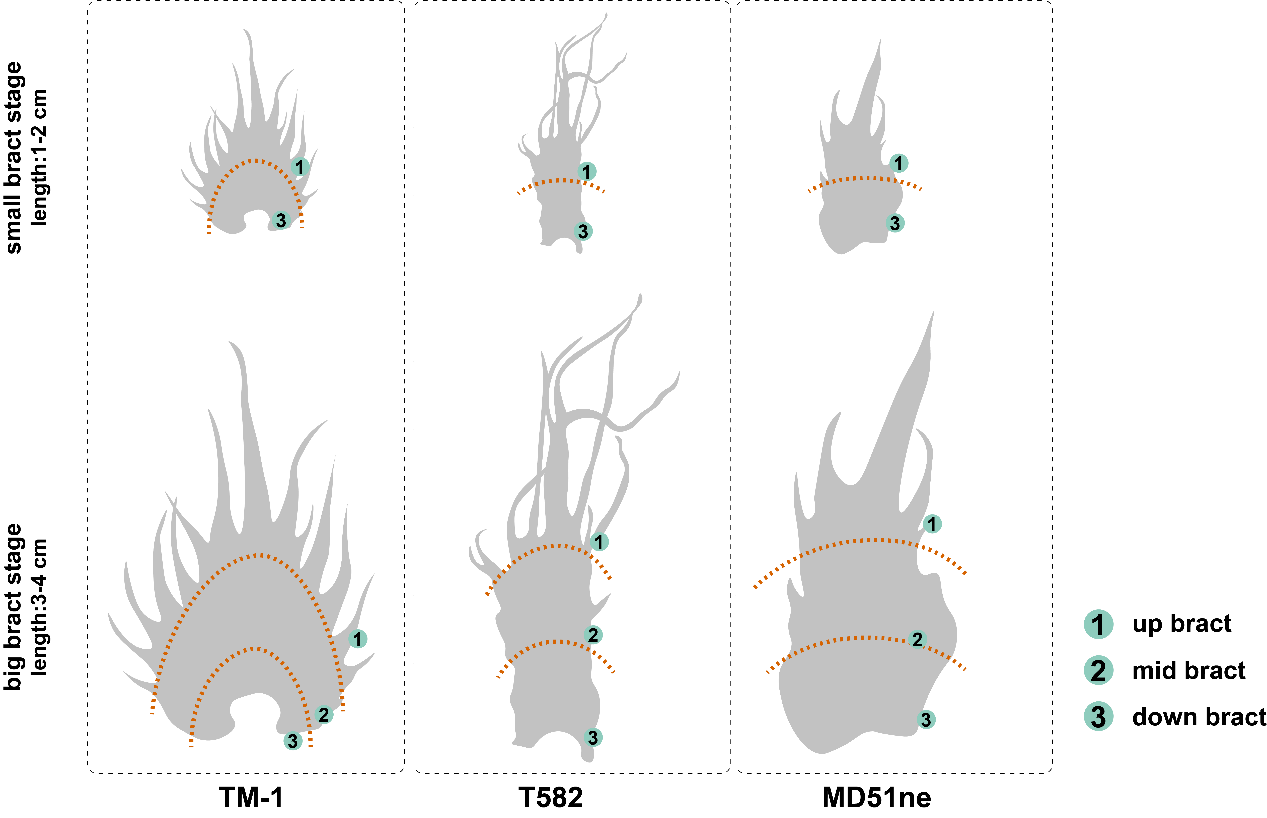


**Figure S13. The diagram of bract cutting and grouping.** The orange dotted line represents the cut position, the number marks the name of each part.


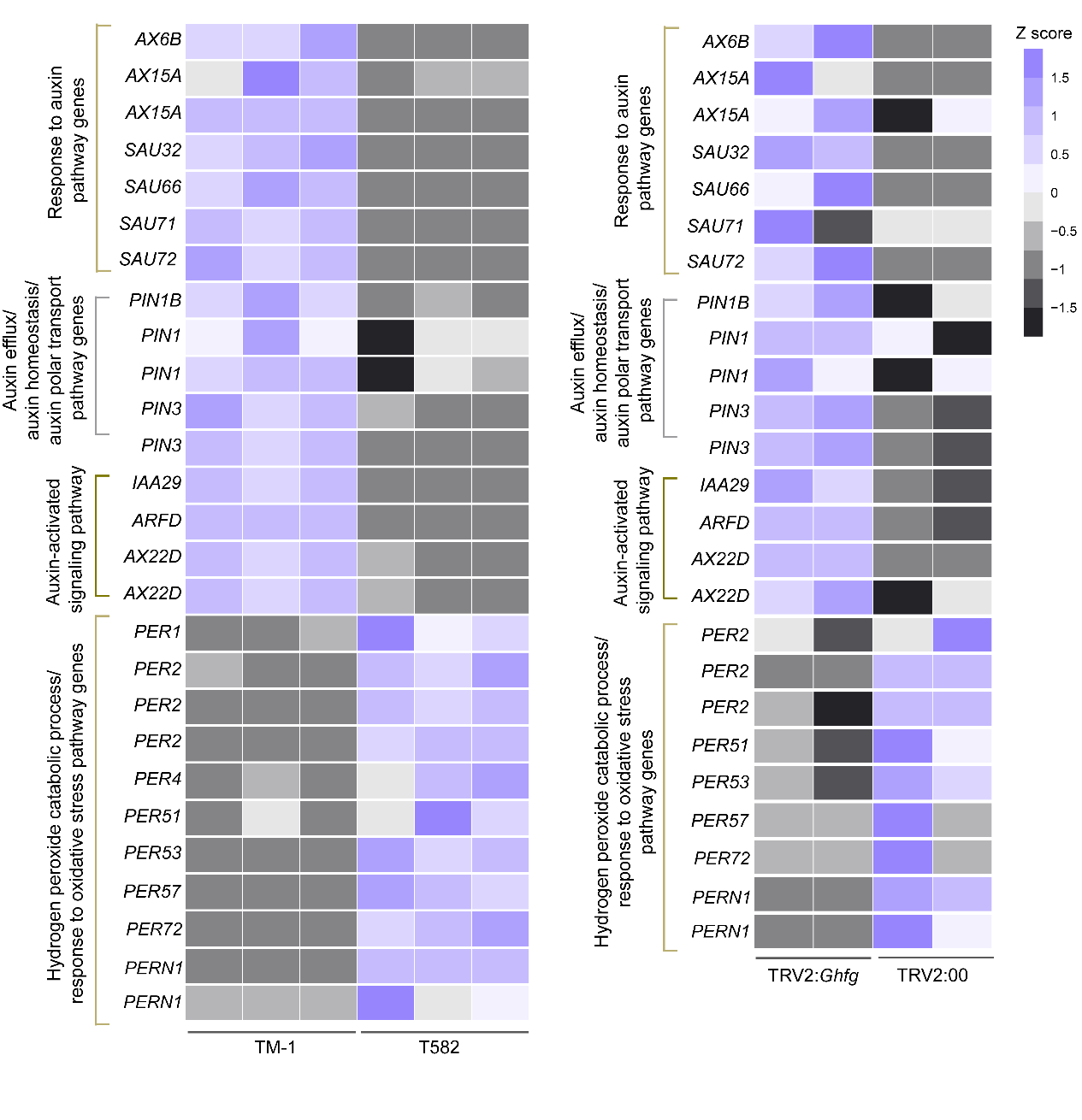


**Figure S14. Heatmaps of genes expression on pathways associated with auxin and reactive oxygen clearance in cotton bracts.** *AX6B*: *auxin-induced protein 6B*; *AX15A*: *auxin-induced protein 15A*; *SAU*: *SAUR-like auxin-responsive protein family*; *PIN1B*: *auxin efflux carrier component 1B*; *PIN*: *PINFORMED*; *IAA29*: *indole-3-acetic acid*; *ARFD*: *auxin response factor 2B*; *AX22D*: *auxin-induced protein 22D*; *PER*: *peroxidase*; *PERN*: *peroxidase superfamily protein.*


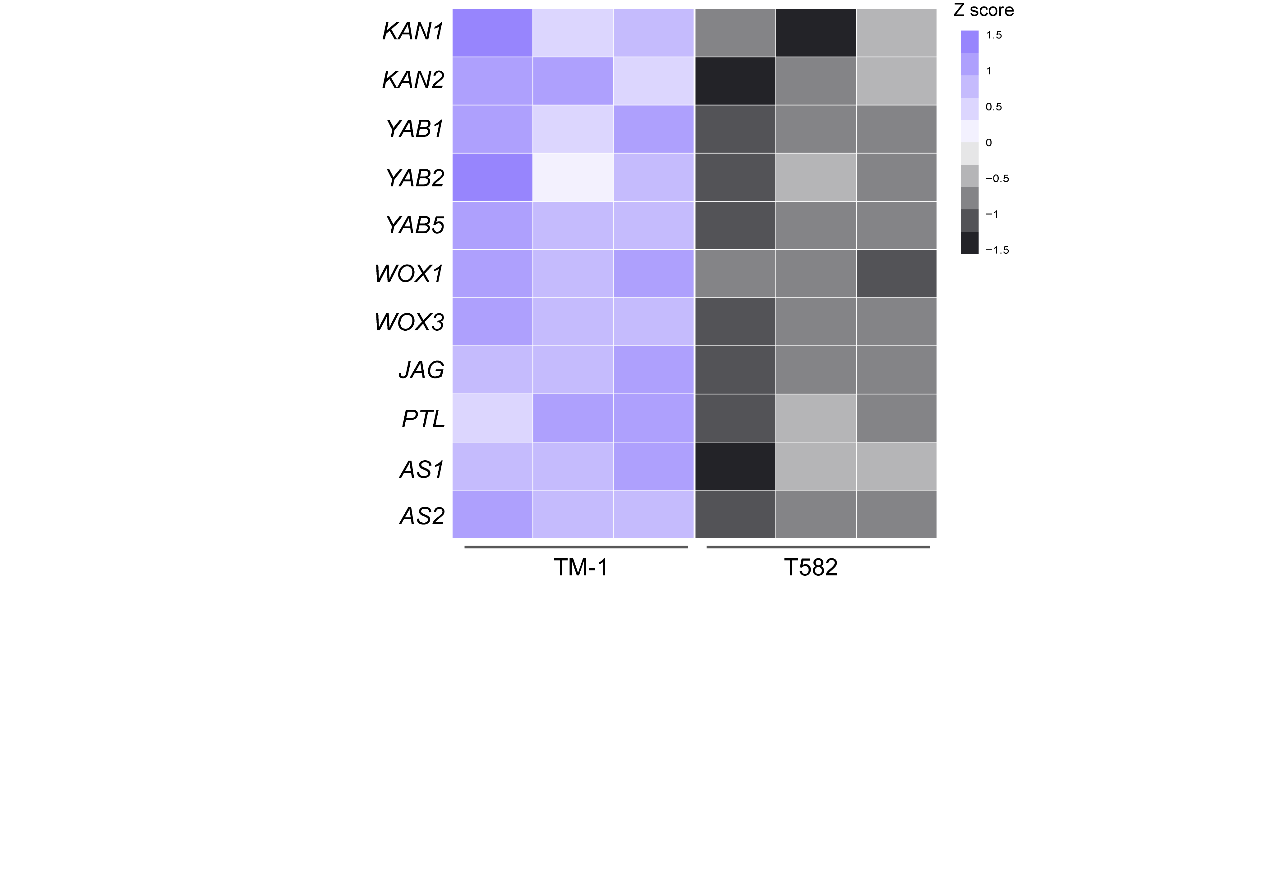


**Figure S15. Heatmaps of genes related to leaf development of cotton bract.** *KAN*: *KANAD*; *YAB*: *YABBY*; *WOX*: *WUSCHEL-RELATED HOMEOBOX*; *JAG*: *JAGGED*; *PTL*: *PETAL LOSS*; *AS*: *ASYMMETRIC LEAVES.*
